# Feasibility of a reconfigured domestic violence and abuse training and support intervention responding to affected women, men, children and young people through primary care

**DOI:** 10.1186/s12875-023-02249-5

**Published:** 2024-01-26

**Authors:** Eszter Szilassy, Caroline Coope, Elizabeth Emsley, Emma Williamson, Estela Capelas Barbosa, Medina Johnson, Anna Dowrick, Gene Feder

**Affiliations:** 1 Centre for Academic Primary Care, Bristol Medical School (Population Health Sciences), University of Bristol, Canynge Hall, 39 Whatley Road, Bristol, BS8 2PS UK; 2 Bristol Medical School (Population Health Sciences), University of Bristol, Canynge Hall, 39 Whatley Road, Bristol, BS8 2PS UK; 3 IRISi, Bristol, UK; 4 Nuffield Department of Primary Care Health Sciences, University of Oxford, Oxford, UK

**Keywords:** Domestic violence and abuse, Women and men victim-survivors, Women and men perpetrators, Children and young people, Training, Intervention, General Practice, Primary Care, Feasibility study

## Abstract

**Background:**

Identification in UK general practice of women affected by domestic violence and abuse (DVA) is increasing, but men and children/young people (CYP) are rarely identified and referred for specialist support. To address this gap, we collaborated with IRISi (UK social enterprise) to strengthen elements of the IRIS + intervention which included the identification of men, direct engagement with CYP, and improved guidance on responding to information received from other agencies. IRIS + was an adaptation of the national IRIS (Identification and Referral to Improve Safety) model focused on the needs of women victim-survivors of DVA. Without diminishing the responses to women, IRIS + also responded to the needs of men experiencing or perpetrating DVA, and CYP living with DVA and/or experiencing it in their own relationships. Our study tested the feasibility of the adapted IRIS + intervention in England and Wales between 2019–21.

**Methods:**

We used mixed method analysis to triangulate data from various sources (pre/post intervention questionnaires with primary care clinicians; data extracted from medical records and DVA agencies; semi-structured interviews with clinicians, service providers and referred adults and children) to assess the feasibility and acceptability of the IRIS + intervention.

**Results:**

The rate of referral for women doubled (21.6/year/practice) from the rate (9.29/year/practice) in the original IRIS trial. The intervention also enabled identification and direct referral of CYP (15% of total referrals) and men (mostly survivors, 10% of total referrals). Despite an increase in self-reported clinician preparedness to respond to all patient groups, the intervention generated a low number of men perpetrator referrals (2% of all referrals). GPs were the principal patient referrers. Over two-thirds of referred women and CYP and almost half of all referred men were directly supported by the service. Many CYP also received IRIS + support indirectly, via the referred parents. Men and CYP supported by IRIS + reported improved physical and mental health, wellbeing, and confidence.

**Conclusions:**

Although the study showed acceptability and feasibility, there remains uncertainty about the effectiveness, cost-effectiveness, and scalability of IRIS + . Building on the success of this feasibility study, the next step should be trialling the effectiveness of IRIS + implementation to inform service implementation decisions.

**Supplementary Information:**

The online version contains supplementary material available at 10.1186/s12875-023-02249-5.

## Background

Domestic violence and abuse (DVA) is a public health challenge [1–4] with vast social and economic costs [5, 6]. It results in increased use of health and other services by people affected through victimisation and exposure and carries a significant risk of death. Primary care is a key location for interventions to prevent DVA and improve health outcomes for adults and children. In addition to providing a safe and confidential place for DVA disclosure, primary care can enable crucial access to specialist advocacy support. This specialist support in the UK largely comes from third sector DVA agencies. They have a crucial role in prevention, early identification, and provision of support [7].

DVA interventions so far have prioritised women, who are disproportionately affected in prevalence and severity [8, 9], including domestic abuse related death [10]. Identifying women survivors in primary care and referring them to specialist support is effective (in terms of recorded referrals, a proxy for patient’s benefit) and cost-effective (from a societal and health service perspective) through provision of DVA training linked with local DVA support [11, 12]. The leading UK service model is IRIS (Identification & Referral to Improve Safety), an evidence-based training and advocacy support programme addressing the needs of women DVA survivors. IRIS is increasingly recognised and commissioned in England, Wales, the Channel Islands and Northern Ireland, but is not consistently or sustainably available across the UK (trained > 12% of all general practices). IRIS nurtures greater health service engagement with DVA by linking primary care to the third sector response to violence against women, via the DVA agency-employed advocate educator [11]. Success in identifying women through IRIS is growing, but men survivors and children/young people (CYP) who witness/experience DVA are rarely identified in primary care and referred for specialist support. The mental and physical health impact across the life course of CYP [13, 14] and on men survivors [10, 15–17] thus remains neglected in the primary care DVA response.

Recognising the needs of a wider range of patient groups and to address these gaps, we collaborated with IRISi (UK social enterprise) and DVA agencies to develop and pilot IRIS + . IRIS + is an adapted IRIS programme responding to the diverse needs of all patient groups. Building on the IRIS training and advocacy support programme, IRIS + offers a coordinated whole-systems approach for DVA training and advocacy interventions. IRIS + adds to the existing IRIS model by expanding clinical focus, patient care pathways and specialist advocacy support to men and children. While the IRIS programme prioritises the needs of women survivors of DVA, IRIS + , without diminishing the focus on women, also responds to the needs of men experiencing or perpetrating DVA, and CYP living with DVA and/or experiencing it in their own relationships.

Evidence from our previous study [18–20] informed the adaptation of the IRIS + intervention. The current study, conducted between 2019–21, tested the adapted IRIS + intervention for feasibility and prospective cost-effectiveness [21] using mixed method evaluation.

## Methods

### Intervention reconfiguration

The IRIS + training and support intervention [19] was built on and expanded the IRIS training and advocacy support programme. The adapted IRIS + intervention was reconfigured to enhance the identification and referral of men and children directly and via reports received from other agencies. It also aimed to extend the professional scope of the training to include key health care professionals linked with general practice, training them together with core primary care teams and enabling them to make referrals directly.

**Fig. 1:**
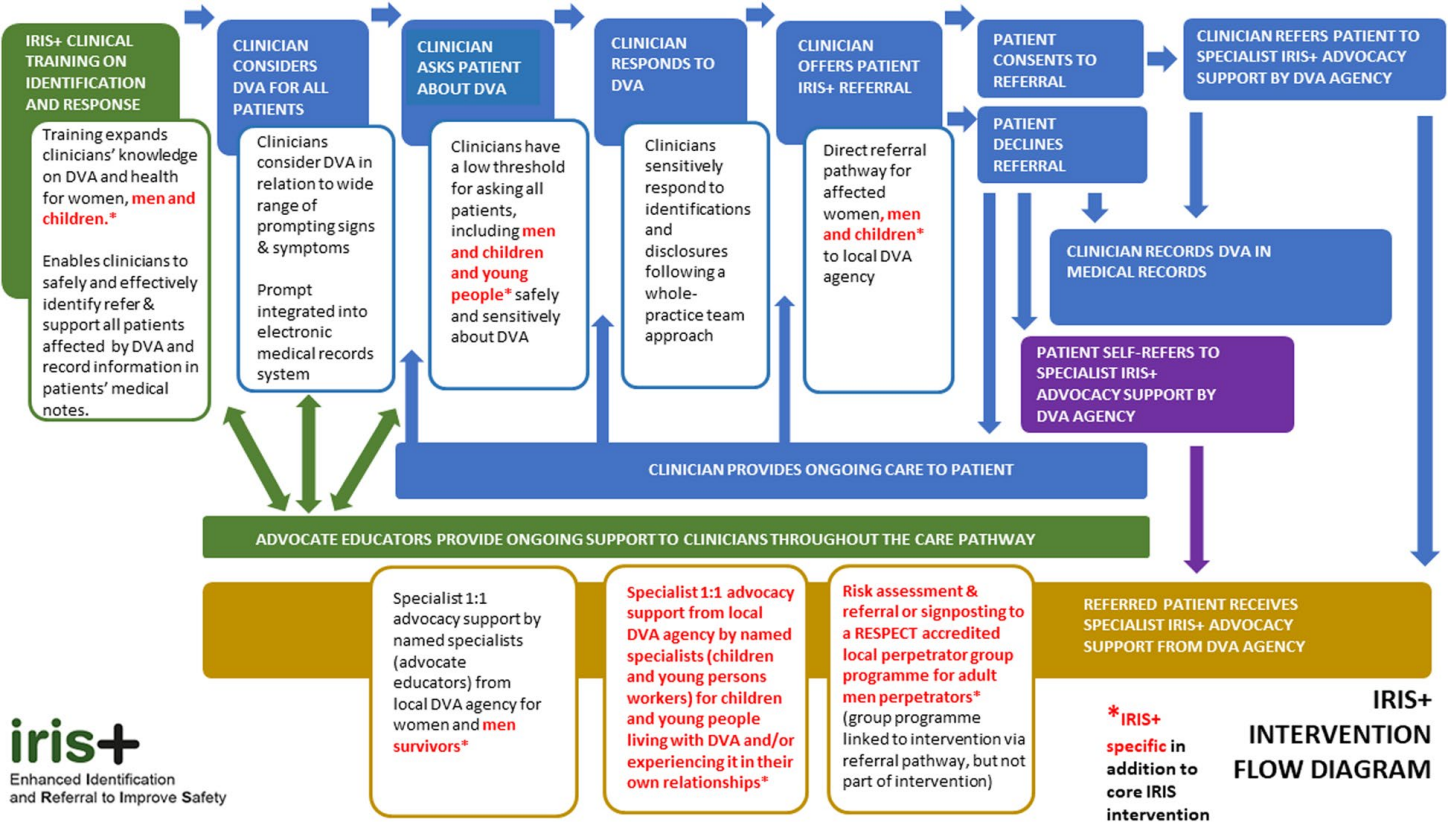
IRIS + intervention flow diagram

The intervention comprised the following components: (i) clinical training to primary care teams, including clinicians affiliated with local primary care teams (i.e. health visitors, substance abuse liaison workers, and other allied health professionals based at the practice) about DVA among women, men and CYP; an online resource for clinicians alongside the training; and a medical records prompts system; (ii) direct referral pathway for affected women, men and CYP to a named specialist from a local DVA agency, called an advocate educator (AE); (iii) specialist 1:1 advocacy support by the AE for women and men survivors and for CYP living with DVA and/or experiencing it in their own relationships; (iv) risk assessment and signposting/referral to a local perpetrator group programme for adult men perpetrators (Fig. 1).

The clinical training was co-delivered by an AE, a social worker specialised in children and DVA, and a local IRIS + clinical lead (practising general practitioner (GP) with an expertise in DVA). Advocacy support for adult patients was provided by the AEs. CYP were supported by the children and young persons’ workers (CYPW). The AEs and the CYPWs were based in local voluntary sector DVA agencies (IRIS + hubs) in the intervention sites. They received referrals from clinicians and provided expert advocacy to referred women and men adults and CYP affected by DVA. The clinical training was adapted to be relevant locally.

### Practice recruitment and intervention delivery

IRIS + was tested in two urban areas in England and Wales in a mixture of IRIS-trained and non-IRIS trained general practices. Recruitment of practices was informed by practice size, socio-demographics characteristics of the population served, DVA referral activity (for IRIS-trained practices), and practices’ availability for training and research.

Three non-IRIS practices received two sessions of two-hour face-to-face interactive training intervention and four IRIS-trained practices received one two-hour training session. Training was delivered between June 2019-Jan 2020. The training intervention included a one-hour information session for reception and administrative staff and an additional brief (up to half an hour) online reminder and question and answer session during a clinical practice meeting during the IRIS + intervention period. The IRIS + intervention was delivered between June 2019 and June 2021. This included a direct patient referral pathway from general practices to the IRIS + hubs between June 2019 and December 2020. Referred patients received DVA advocacy support from the IRIS + hubs for up to six months.

### Evaluation of feasibility

We used a range of methods to assess the feasibility of the reconfigured IRIS + intervention. This included (i) measuring change in clinicians’ practice and behaviour through a pre/post questionnaire (PIM +) developed from the PIM (PROVIDE Intervention Measure) questionnaire [22]. Clinicians undertaking the training were asked to complete the online survey before the training (Additional file 1) and again after 12 to 16 months (Additional file 2); (ii) DVA identification data extracted pre-and post-intervention from the electronic medical records (EMR) of the participating practices for a period of 18 months after the delivery of the first IRIS + intervention to measure clinical DVA identifications during the study period; (iii) IRIS + referral and service support contact data collected from the third sector partner agency post-intervention; (iv) semi-structured interviews with participating clinicians soon after the IRIS + training (Additional file 3) and between six and twelve months later (Additional file 4); (v) semi-structured interviews with professionals delivering and/or facilitating the delivery of the intervention (Additional files 5, 6 and 7); (vi) semi-structured interviews with patients referred soon after their referral/first meeting with the IRIS + AE (adults only) (Additional file 8) and 3–6 months later upon completion of their support intervention (both adults and children) (Additional files 9, 10 and 11). (See research participants’ characteristics below, under Results). Data collection took place between June 2019-August 2021.

Interviews were audio‐recorded, transcribed verbatim, uploaded to qualitative data analysis software (NVivo v.12) and analysed thematically [23] using an inductive approach to develop an initial coding frame. Data in (ii)-(iv) were analysed descriptively in Stata (v. 16.1/MP). Due to small sample size, the study did not aim to draw inferences from quantitative data.

**Fig. 2:**
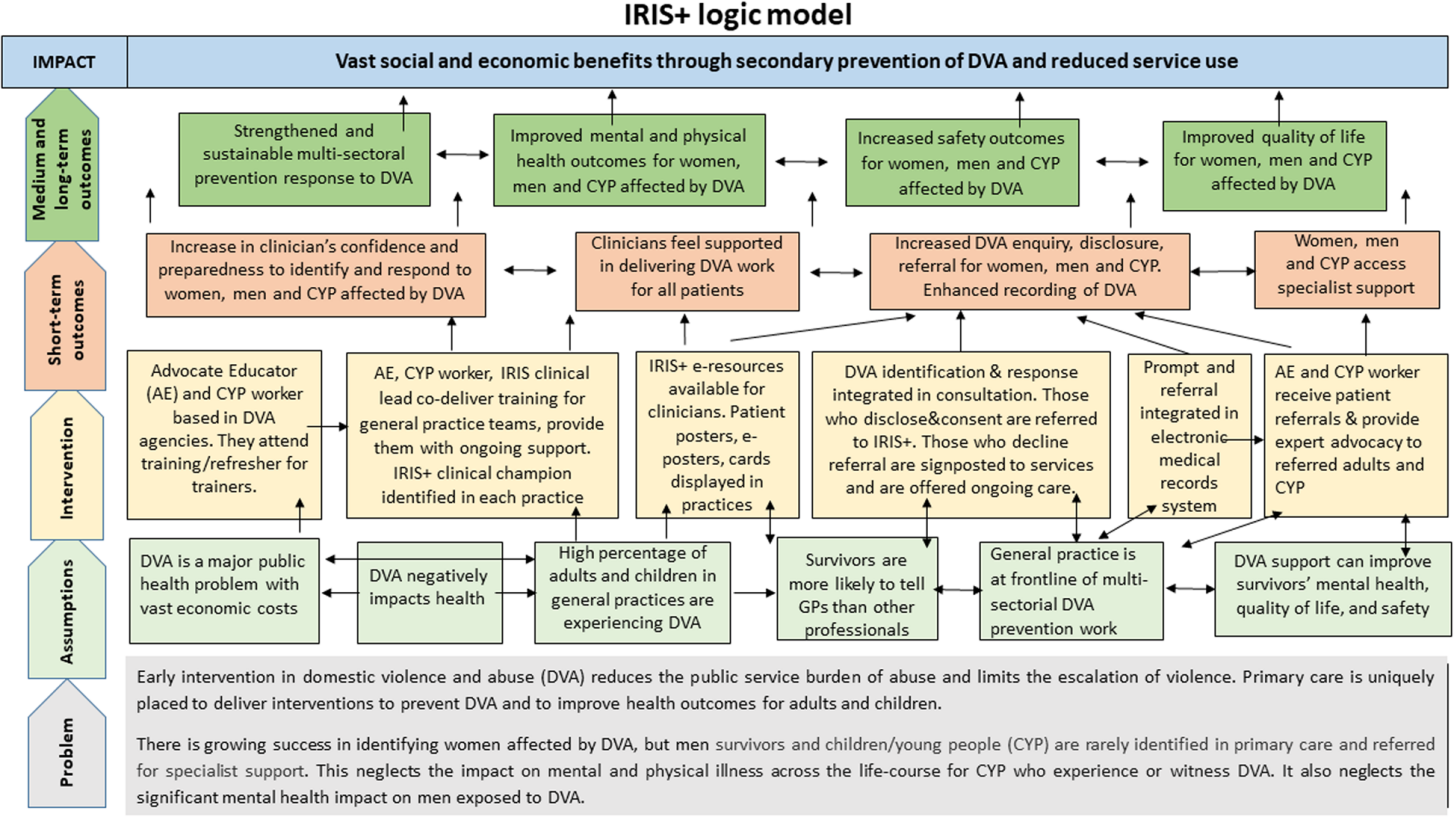
IRIS + logic model

For the mixed method analysis, we used a convergent design where we first independently analysed data sources and then used triangulation to refine our coding frame and map dimensions of feasibility and acceptability to our data [24]. We also explored how our findings mapping to different outcome domains in our logic model (Fig. 2.) contribute evidence for or against the feasibility of the intervention.

Two service user expert groups (women survivor, men survivor) have been closely engaged with the researchers from development of the proposal, through protocol development, writing of participant recruitment materials, development of the intervention, conduct of the study, interpretation and dissemination of findings.

## Results

### Participants’ characteristics

(i) PIM + questionnaire: 94 (65 women, 29 men) of 170 invited primary care clinicians completed the survey at a minimum of one timepoint, with 31 completing the full survey at both time points. Of these, 17 were general practitioners, including three trainees, and 14 were other primary care clinicians based at participating general practices, including practice nurses, nurse practitioners, healthcare assistants, substance abuse liaison workers, urgent care practitioners and health visitors.
(ii) Interviews with primary care clinicians: 16 clinicians (11 women, five men) completed the interview at one timepoint. Of these, eight were general practitioners and eight were other clinicians based at participating general practices. Other primary clinicians included five practice nurses, one substance abuse liaison worker, one urgent care practitioner, one health visitor and one heath care assistant. Eleven clinicians (seven GPs and four other clinicians) completed interviews at two timepoints.
(iii) Interviews with key IRIS + professionals: Eight IRIS + professionals (all women) completed at least one interview. These included three AEs, one DVA clinical lead trainer, one social worker trainer, two CYPWs, one IRIS + support service manager. Two of the eight professionals (two AEs) completed interviews at two timepoints, soon after delivering the clinical training and following the completion of the IRIS + support intervention delivery.
(iv) Interviews with referred adult patients and CYP: Thirty adults (20 women, ten men) completed at least one interview. Twenty-nine adults (19 women, ten men) completed interviews soon after their referral and 14 (eight women, six men) completed interviews upon completion of their support intervention. Twelve adults (six women, six men) completed interviews at two timepoints. Upon support completion, five CYP aged 8–16 (one girl, four boys) completed semi-structured interviews.

### Identifications and referrals

#### Preparedness to respond to DVA

Clinicians’ PIM + survey responses completed at both time points on perceived preparedness indicated that the intervention had led to improvements in all areas of clinical practice. Participating clinicians’ perception of preparedness consistently improved in relation to responding to the needs of all patient groups, including female and male survivors, CYP and their parents. Clinicians felt more prepared to ask questions, identify signs and symptoms of DVA and provide appropriate response to disclosures (Table 1.).

**Table 1:** Change in clinicians’ self-reported preparedness to respond to DVA

**PIM + questionnaire domains**	**n**	**T1 mean score**	**T2 mean score**	**Median change**	**95% CI**	**Wilcoxon signed- rank test P-value**
**Ask about DVA**						
Female victims	31	3.2	4.1	1.0	[0.5, 1.5]	0.0003
Female perpetrators	31	2.0	3.3	1.5	[1.0, 2.0]	0.0000
Male victims	31	2.6	3.8	1.0	[1.0, 1.5]	0.0000
Male perpetrators	31	2.1	3.5	1.5	[1.0, 2.0]	0.0000
Parents	31	2.7	3.8	1.0	[0.5, 1.5]	0.0001
Children and young people	31	2.7	3.7	1.0	[0.5, 1.5]	0.0005
**Identify signs and symptoms of DVA**						
Female victims	31	3.3	4.1	1.0	[0.5, 1.0]	0.0002
Female perpetrators	31	2.1	3.3	1.0	[1.0, 1.5]	0.0000
Male victims	31	2.7	3.8	1.0	[1.0, 1.5]	0.0000
Male perpetrators	31	2.3	3.5	1.0	[0.5, 1.5]	0.0001
Parents	31	3.0	3.7	0.5	[0.5, 1.0]	0.0003
Children and young people	31	3.0	3.9	1.0	[0.5, 1.0]	0.0001
**Respond to initial disclosure of DVA**						
Female victims	31	3.2	4.3	1.0	[0.5, 1.5]	0.0000
Female perpetrators	31	2.0	3.8	2.0	[1.5, 2.5]	0.0000
Male victims	31	2.7	4.1	1.5	[1.0, 2.0]	0.0000
Male perpetrators	31	2.2	3.9	1.5	[1.0, 2.0]	0.0000
Parents	31	2.9	4.1	1.0	[1.0, 1.5]	0.0000
Children and young people	31	2.9	4.1	1.0	[1.0, 1.5]	0.0000
**Refer**						
Female victims	31	3.3	4.4	1.0	[0.5, 1.5]	0.0001
Female perpetrators	31	1.8	4.0	2.5	[2.0, 2.5]	0.0000
Male victims	31	2.6	4.3	1.5	[1.0, 2.0]	0.0000
Male perpetrators	31	2.1	4.1	2.0	[1.5, 2.5]	0.0000
Parents	31	2.7	4.2	1.5	[1.0, 2.0]	0.0000
Children and young people	31	2.9	4.2	1.0	[1.0, 2.0]	0.0000
**Record information about DVA**						
Female victims	31	3.3	4.2	1.0	[0.5, 1.0]	0.0001
Female perpetrators	31	2.7	4.0	1.5	[1.0, 1.5]	0.0001
Male victims	31	3.1	4.1	1.0	[0.5, 1.5]	0.0001
Male perpetrators	31	2.7	4.0	1.5	[1.0, 1.5]	0.0001
Parents	31	3.1	4.0	1.0	[0.5, 1.5]	0.0002
Children and young people	31	3.1	4.1	1.0	[0.5, 1.5]	0.0000
**Provide ongoing support**						
Female victims	31	3.1	4.0	1.0	[0.5, 1.5]	0.0002
Female perpetrators	31	1.9	3.5	1.5	[1.5, 2.0]	0.0000
Male victims	31	2.7	3.8	1.0	[1.0, 1.5]	0.0000
Male perpetrators	31	2.0	3.5	1.5	[1.0, 2.0]	0.0000
Parents	31	2.7	3.7	1.0	[1.0, 1.5]	0.0000
Children and young people	31	2.8	3.9	1.0	[1.0, 1.5]	0.0000

*This table reports the number of paired observations; mean preparedness score [range 1–5] at time points 1 and 2; the Hodges-Lehmann estimate of the median change and its 95% confidence interval (CI); and the Wilcoxon Signed Ranks Test of the change (T2-T1) in median score*

Interviews with clinicians corroborated the survey findings. Those participating in the intervention reported increased knowledge and confidence in asking all patients about their experiences as a result of completing the clinical training and working within the IRIS + referral and support structure. As a lead nurse (PN5) explained:

Certainly with the younger girls, it's encouraged me to talk to people about relationships a bit more and what's okay and what isn't okay in relationships.

Although GPs have ample experience discussing sensitive issues with patients, the training gave clinicians confidence to ask them specifically about DVA. A practice nurse found the clinical training helpful in hearing practical examples, from both the trainers and the rest of the clinical team, of what follow-on questions to ask from difficult conversations or disclosures:

Things like, “Do you think anyone else doesn’t feel safe when you get angry like that?” Just to move that conversation on. (practice nurse, PN4).

I do feel more comfortable about asking people about domestic violence […] it’s a bit like asking people about suicide, basically. You have to do it, and you just get in the habit and you find your way of doing it. (GP6).

Knowing that there is a way to ‘prescribe’ help, in the form of specialised DVA support that can be put in place via the IRIS + referral, enabled clinicians’ confidence to ask patients about DVA:

It just felt nice that I was able to help these people. Nice to be able to offer them something. […] It's a breath of fresh air. (GP3).

They also noted their clinical teams’ eagerness to be more prepared to support patients affected by DVA. A social worker trainer (SW1) described that clinical teams were keen to discuss ‘*how to support children who were clearly having a difficult time, but were not ever going to be accepted for referral at children’s social care level and, how to introduce the topic of domestic violence and abuse, when you could maybe talk to a child by themselves.’*

#### Features of identifications and referrals

Clinicians’ readiness to respond translated into DVA referrals. In total 256 adults (227 women and 29 men) and 44 CYP were directly referred from seven general practices to the IRIS + hubs from the end of June 2019 to the end of December 2020. Although 44 CYP were referred directly to IRIS + , there were an additional 213 CYP identified as potentially being exposed to DVA by being listed on the adults’ referral forms. The rate of referral for women in the study period was more than double than that of the original IRIS trial: 21.6 per year per IRIS + intervention practice compared to 9.29 per year per IRIS intervention practice [11]. IRIS + referrals included mostly women survivors. About ten percent of all referrals were for men and 15 percent of all referrals were for direct referrals for CYP. Referrals were made for different types of DVA, including coercive control and psychological abuse, and included referrals for current and historical DVA. Data on the reasons for referral (type of abuse, etc.) were incomplete, but our interviews with adult survivors revealed different forms of abuse, and included many accounts of multi-victimisation where multiple forms of abuse have been experienced. Referrals were made for a wide range of age groups and ethnicities. Over two-thirds of referred women and directly referred CYP and nearly half of referred men were directly supported by the IRIS + service. Those not supported by IRIS + either declined the offer to receive support and/or were signposted to other services. In addition, many CYP have also received support indirectly via the referred parents (Table 2).

**Table 2:** Referral and IRIS + support

	Referred by GP				Supported by IRIS +		
	Number of referrals (% of total referrals)				Number of patients supported (% of directly referred in corresponding patient groups)		
	Adult		CYP		Adult		CYP
	Women	Men	Direct referral for CYP or self-referral	Listed on adults’ referral form	Women	Men	
	227 (75.6%)	29 (24 survivors, 5 perpetrators) (9.7%)	44 (14.7%)	213	157 (69.2%)	12 (41.4%)	30 (68.2%)
Total	300			213	199		

Total number of DVA patient referrals from IRIS + general practices to DVA services for the period 20/06/2019 to 31/12/2020 and total number of referred patients supported by the IRIS + hubs

Most referrals were made either before or after the COVID-19 national lockdown periods (23 March to 23 June 2020). When comparing pre-pandemic (1 June 2019 to 22 March 2020) and pandemic (23 March 2020 to 31 December 2020) time periods of similar time lengths within the IRIS + intervention, the latter period saw a one third reduction in IRIS + referrals from study general practice teams, which corresponds to findings from other studies [25]. We found no marked change in DVA identifications in the EMR comparing the pre-pandemic and the pandemic intervention periods in the four GP practices supporting the EMR data collection. In the pre-pandemic period, however, DVA was more frequently identified in a patient consultation than through third party information from reports sent to general practice from external organisations including the police, children’s social care services, MARAC, DVA services, and A&E (107 versus 48). This was reversed in the pandemic period when DVA was identified more via third party documents than through patient consultations (86 versus 70). During the pandemic period there was also a nearly 80% increase in third party DVA identifications within the total number of identifications, such as from the police (53 DVA identifications versus 17), who subsequently notified general practice about DVA (Table 3.).

**Table 3:** Identification and referral in pre-pandemic and pandemic periods

	**Pre-pandemic period**	**Pandemic period**
**Total number of DVA identifications by general practice recorded in EMR**	161	169
Identifications via patient consultations	107	70
Identifications via reports received from third parties	48	86
Police	17	53
Other (A&E, children’s social care services, MARAC, etc.)	31	33
**IRIS + referrals recorded in EMR**	66	43

*DVA identifications and referrals in pre-pandemic (1 June 2019 to 22 March 2020) and pandemic (23 March 2020 to 31 December 2020) periods in four GP practices*

The reconfigured intervention enabled a more effective use of third-party information for the identification of patients experiencing/perpetrating DVA. One of the clinicians articulated the value of IRIS + in supporting DVA identification, comparing IRIS + to usual care:

Having the reminder that the service is there and that we should be using it, and if certain stuff comes through to us and the information from other parties comes through to us that actually allows, than just kind of clicking and saying, “Yes, that’s fine,” it would be a bit more sort of, “Okay, what can we do about this?” (GP4).

#### Whole team approach to identification and referral

We found that pre-training, GPs had higher self-reported preparedness scores than other clinicians across the range of DVA response behaviours measured, and particularly regarding female survivors and information recording. Post-training there was a marked increase in scores for the knowledge and confidence to respond to DVA among other clinicians, but this did not translate into changes in their clinical practice (i.e. making DVA referrals directly themselves). General practitioners were the principal referrers of patients across all patient age groups and genders compared to other clinicians: of the 300 referrals, 269 came from GPs, 14 from other clinicians, and 17 from self-referrals, following a GP visit.

Continuity of care was seen by clinicians as an important prerequisite for effective identification of DVA in general practice. GPs felt that because of their familiarity with a list of families, they were well placed to make DVA referrals:

It makes sense [GPs referring], because we work on a list. You have your list of patients who are under you generally, so it makes sense in terms of joining things up [for nurses] to go to the GP who might know that patient the best. (GP6).

We might see a patient once and then never again. I guess these things might be a bit more difficult to pick up on a quick popping-in to have their chest listened to or whatever it might be. (Urgent care practitioner—UCP1).

Despite GPs being the primary referrers, clinicians emphasized that effective responses to DVA, including patient referrals, were enabled by a collaborative approach involving the primary care team as a whole. A substance abuse liaison worker (SALW) said that being able to *‘spread the weight’* was one of the most helpful aspects of the intervention:

I know that I can go to my GP and have that conversation. […] So, there is a process, so, you know, with this sort of stuff, sometimes it feels really heavy […] and knowing that I can refer to IRIS + is always great to know. (SALW1).

Interview participants acknowledged the value of training primary care teams together and the importance of a supportive team environment enabling information exchange and peer-support:

The fact that the whole team were pulled together. That’s quite a rare event for us, actually, to achieve that… I’ll hear what others have to say and how others are comfortable to frame these questions and what seems to work well. […] That will then be more likely to come forward into my mind when I’m sat facing somebody who says something that might not be typical for my usual review about their diabetes condition as such. (PN4).

The collective team approach supporting effective primary care pathways to IRIS + support also involved non-clinical administrative support staff in the process of DVA identification and care. An IRIS + AE described that, following training, the administrative team were:

really empowered that they can have a role […] I was saying to them, "You're the ears and you're the eyes of the surgery. You're seeing them [patients] for a longer period of time in the waiting room. " […] When I left, they were all like, "We're going to go out today and we're going to be the ears of the surgery." (AE1).

Although all clinician groups felt enabled to and responsible for identifying patients affected by DVA, many non-GP clinicians regarded referral-making as being outside of their role boundaries. ‘*[I]f I do feel someone is in trouble’,* said a health care assistant*, ‘[I] report it, talk to the doctors’* (HCA1). As an AE (AE2) noted, *‘they [GPs and practice nurses] have that conversation, but the referrals themselves do tend to come from GPs’.* ‘*Traditionally, referrals to all other sources would come from the GP’,* described a GP* (*GP7). Other clinicians also acknowledged that although they see DVA care as within their competence, they would typically take their concerns about patients affected by DVA to a general practitioner and defer the actual act of referral to them:

I had a patient, an alcohol patient, who came to see me. […] She kind of opened up to me, not the doctor, and I discussed it with the doctor and, with an agreement, she went back to her doctor […] I thought coming from a GP [DVA referral] would probably hold more weight. (SALW1).

Professionals both delivering and receiving the IRIS + intervention recognised that ‘*nurses are not as used to making referrals’* (clinical lead, CL1). Differences in the nature of the GP and non-GP clinical encounters with patients were used to explain differences in referral-making.

When you're going in to see a nurse, you're going in because you're having a blood test, you're having a smear test or you're having something very specific done. Whereas with GPs, there's that element more of having the chat, even though it's a very brief one: "How are things?" I wonder if that's got something to do with it. (IRIS + AE—AE2).

### Reaching men

#### Impact of IRIS + on clinical responses to men

Primary care clinicians’ increased awareness of DVA affecting men and their confidence to use the direct DVA referral pathway to the IRIS + service led to the identification and referral of men affected by DVA. Most referrals (23) were made for men survivors and a small number (6) were made for perpetrators. All of the 28 men referred to IRIS + by a clinician were referred by their GPs. Only one man (survivor) self-referred, indicating the effectiveness of active referral compared to signposting. Referrals for men were made in both previously IRIS-trained and non-IRIS-trained general practices, but most referrals (19) came from IRIS-trained practices.

Clinicians explained that the availability of a quick direct referral pathway for both men survivors and perpetrators and the responsiveness of the IRIS + service simplified the referral process. Another GP explained that knowing that there was a service that would support people that she was *‘worried about, or they were worried about their own actions, was a really good thing’.* Expanding on her experience of referring, she said:

I did make a referral for somebody, a gentleman, who was worried about the way that he was treating his family and the way that they were scared of him. I was able to actually implement what I learned which was really nice. (GP4).

Not having to label survivors or perpetrators on the referral form and the availability of ongoing support for clinicians from the AEs facilitated the process. It also enhanced clinicians’ confidence to talk to men about their experiences of abuse:

I was like, “Okay, cool, that’s really easy. I can put that in my phone or in my notes, and then if I've got any worries, I can just talk to [AE],” so that’s really good. (GP2).

Clinicians agreed that it takes time to embed learning about DVA in practice, and the development of skills to routinely ask difficult questions and refer patients requires repeated efforts and practice. The lack of ‘practice’ and ease asking men about their potentially abusive behaviour was reflected in the small number of referrals for perpetrators.

*With the perpetrators you just don’t come across them as often. So, you’re not really getting the practice of learning how to broach the topic, and it’s such a sensitive topic as well.* (GP6).

#### Men survivors’ experiences of identification and referral

Men survivors supported by the IRIS + service and participating in the interview study were all referred to IRIS + during or following a face-to-face GP visit. They spoke positively about their experiences of disclosure and referral to IRIS + :

She [GP] was great. […] she referred me to IRIS + and said, “I feel that you have got the right credentials, for what’s been going on.” Because, again, it’s not something that’s ever really crossed my mind. (adult man patient 2).

I’ve had some dark thoughts. I have thought about ending my life. Yes, so the doctor’s been quite good. […] She said, “I can see you’re down; I can see you’re low.” She put me in contact with [AE]. (adult man patient 3).

As one of the AEs supporting men survivors recounted:

He [client] was so appreciative of the GP, he was like, “That GP just knew, and they asked that question. As soon as that GP asked that question, I was then able to say what happened. But if it wasn’t for that GP asking that direct question, I would probably still be stuck now.” (AE1).

Similarly, another participant recalled the ‘turning point’ when he had decided to seek help from general practice in relation to chronic mental health difficulties which, in turn, led to DVA identification and IRIS + referral:

Because I speak out and that’s what saved me today, because if I keep it inside me, I would be a dead person. (adult man patient 4).

#### Overcoming barriers to DVA identification and referral of men

The interviews with patients and clinicians also shed light on specific barriers to identifying and referring men to the IRIS + hub. The two most frequently discussed barriers to overcome during the process of disclosure included the erosion of continuity of care and the strong societal perceptions about masculinity. Although they influenced patient engagement in different ways, they were both undermining opportunities for disclosure and identification for men in the primary health care setting.

Continuity of care for men survivors participating in the study was typified by an ongoing trusting doctor-patient relationship with the same general practitioner. It meant the time needed to develop rapport with a clinician and build trust and courage to disclose *‘because it's hard to tell people’* (adult man patient 6).

I’d always arrange with my GP to have the last appointment of the day and he’d stay half an hour, forty minutes longer. You know, after obviously the patients have finished, I’d always be the last one because he knew that I needed to talk. […] It helped me a lot because I felt quite low. (adult man patient 7).

Whenever I make an appointment, I’d get an appointment with him […] It’s definitely a big help to see the same doctor. (adult man patient 8).

Adult man patient 7 also explained how he insisted on not wanting to change general practice despite the surgery being at a major distance from his new, safe address that he had been offered through support from IRIS + :

I'm not within their area, but they’ve kept me on their book, so I can still use the same doctor […] otherwise, it means changing my doctor to where I am at the moment, and I don’t think I could have dealt with another person with the trust.

For clinicians, the increasing lack of continuity of care meant the difficulty of building a cumulative picture of concern, in terms of both the relational and the informational components of care. According to a practice nurse *(*PN4), ‘*That close relationship of knowing your regular patients is quite threatened by the whole push towards larger and larger practices’.* Clinicians felt that the inability to establish an ongoing patient-clinician relationship may largely contribute to possible under-detection.

A GP working in a GP cluster described the difficulty of fragmented care in terms of DVA detection in a multi-site setting:

We’re not exclusively based in one [site] all the time, so sometimes you’ll go a long time between seeing people. I must admit, probably previously, I hadn’t necessarily been aware of what was going on […] it kind of happened, those lightbulb moments, in regards to that explains some of it, you know, chronic health seeking behaviours. (GP7).

A shift to remote consulting and triage, which has remained part of primary care since the pandemic, further challenged continuity of care, patient access and opportunities of detection according to both clinicians and men patients:

I do worry that things aren’t being picked up […] when we have to do so much virtually. (GP4).

In relation to receiving support from his GP during the pandemic, a man survivor noted that he was ‘*not really keen on phones*’.

I prefer face to face because I like to tell from their [GP’s] facial expressions how things are going. (adult man patient 6).

Men’s fear of disclosure was closely associated with external and internal pressures dictated by stereotypes and expectations related to masculinity. ‘*Things like this, men don’t talk about’*, said one of the men participants (adult man patient 4). *‘Because you’re a man, you’re supposed to be strong*’, noted another, adding: ‘*Physically, I’m strong…’* (adult man patient 7). Challenges to masculinity diminished men’s confidence to acknowledge and express their feelings about their experiences of abuse. The stigma of being a man victim of DVA, the fear of not being believed, and being falsely accused of perpetration of DVA made them reluctant to seek support:

Because you're a man, you don’t realise you're being abused. So, yes, it’s quite hard. Because you are a man, you don’t want to be…I suppose you don’t want to be less of a person. (adult man patient 2).

Masculine identity as a barrier to acknowledging abuse or a victim status both in terms of men participants’ personal sense of and their societal interpretations of masculinity was highlighted by IRIS + professionals supporting men survivors. *‘It often takes a lot for a man to go to a GP or to seek medical help’*, mentioned a social worker IRIS + trainer (SW1). An IRIS + support service manager overseeing service implementation noticed that:

For the men they just took that little bit longer before they opened up. I think that’s probably going back, especially the men victims who are slightly older, that men shouldn’t show their emotions. Certainly, when they got to know and trust her [AE], then they were happy to open up and have that emotional support as well. (SM1).

#### *IRIS* + *support offered for men survivors and perpetrators*

Men survivors were supported by the IRIS + intervention for an average of 14 weeks, similar to the average time of support provided for women, although some men were supported by IRIS + for up to six months. Following an initial meeting and risk assessment with the AE, men perpetrators were offered onward referral to a local men perpetrator programme. Men survivors were offered trauma-informed, needs-led emotional and practical one-to-one support on a regular (usually weekly) basis. Pre-pandemic support was predominantly face-to-face, which shifted to a combination of face-to-face, telephone and online meetings during the pandemic period depending on the nature of COVID-19 related restrictions and support needs.

Support included risk assessment, safety planning, emotional support, housing support, legal advice, financial advice, child contact advice, benefits advice, mental health support, and immigration support. Eight of the 29 men (including six survivors and two perpetrators) received parenting and/or child contact related, and/or dedicated support for their children. According to one of the CYPWs:

We do get children referred to us whose main carer is their dad, and their dad is the victim. […] There are those men that are affected by it and they do have children and they do have families. So, for them to access the right support, I think, is really useful for them, in the same way, as it’s quite possible for mothers. (CYPW1).

The whole team approach to delivering care for men experiencing DVA extended to close collaboration between the primary care team and the IRIS + service support team. Ongoing communication between the clinicians supporting affected patients and the AE enabled effective DVA care:

My GP was very supportive. He and [AE] had a couple of meetings together as well, so with the support of both of them, the medical side and the counselling side, together they were both very supportive for me, so that’s played a big part in getting me a bit more confident to do what I needed to do to get here, you know? (adult man patient 7).

#### *Impact of IRIS* + *support on men survivors*

Men participating in the study described how the lack of provisions for men survivors in general was a major barrier in the process of help-seeking. The unavailability of formal support pre-IRIS + diminished their confidence and contributed to their persistent despondency. For all interviewed men, IRIS + was the first service that they were able to access for DVA support:

You're a bloke, you're cast aside. And it’s almost like, everyone says, “It’s alright for me to talk, you need to talk about it, about abuse and things like that,” but who can you turn to, who will believe you? […] If it wasn't for IRIS + , what is there? (adult man patient 2).

Another man described a long ordeal he had gone through before being referred to IRIS + :

I tried several agencies […] they really couldn’t give me any support. Either I was out of area or they hadn’t had the funding or they just dealt with women. […] I was getting panic attacks and I was feeling lonely and just nowhere to turn. You know, just a dark hole. There was no-one I could speak to apart from the GP, but he can only do so much. […] If it wasn’t for my GP I don’t know where I’d be now. (adult man patient 7).

Professionals delivering the intervention felt that they had *‘achieved some really good outcomes for those men’*. According to one of the AEs, IRIS + was *‘very beneficial for men as well. People have left partners, people have been rehoused, there have been legal things put in place. People have gone on to do counselling and built on their self-esteem.’* (AE1).

Men supported by IRIS + spoke about the positive impact of support. Men participating in the study reported improved feelings of safety, and a reduction in abusive behaviours experienced. They also reported improved physical and mental health, wellbeing and confidence. They felt that the emotional and practical support received from the AE had made them feel more confident, more assertive and less alone:

[AE] was fantastic, to be honest, she’ll talk you through how I’m feeling and why I’m feeling that way. […] And I think [AE] helped me understand the situation really, and understand the system. (adult man patient 2).

She [AE] gave me confidence, and now I am better and I go back to work as well a little bit. A little relaxed. And I sleep as well now a little bit better than before. Not a little bit – much better. (adult man patient 9).

### Reaching children and young people

#### Impact of IRIS + on clinical responses to CYP

Of the 44 CYP referred directly to IRIS + , there were 26 referrals made by general practitioners and four were made by other clinicians, including health visitors. There were also 14 self-referrals for either young people wishing to engage with the service or self-referrals made together with (or following) the self-referral of parent survivor. Additionally, there were a large number (213) of CYP listed on the adults’ referral forms as potentially exposed to parental DVA, many of whom have received IRIS + support indirectly via the referred parents. All general practices referred CYP.

The questionnaire with clinicians indicated that the IRIS + training had led to significant improvements in skills, confidence and knowledge in identifying asking, responding, referring, recording and supporting CYP and their parents affected by DVA. Clinicians’ preparedness improved in all domains of DVA care for CYP. These included increased awareness about how DVA may impact on CYP’s health and confidence to ask about DVA (Table 1.).

It just really makes you completely aware to watch for little signs and symptoms, especially with people coming in with children, whether the children are a little bit vulnerable and needy. It just makes you look at people in a different light, I think, really (HCA1).

Clinicians understood that, with IRIS + service in place, they could now convert their improved skills and self-efficacy to recognise that CYP might benefit from the service. As one GP explained:

I felt that we were quite privileged as a practice to have the IRIS + , because it just sounded like a really good service. […] My role is just to maybe recognise that someone might need that service. I found that helpful. (GP6).

#### Filling a service gap for CYP

Clinicians and service providers thought that IRIS + had filled a service gap. They believed that it was a particularly valuable resource in identifying CYP who fell below child protection service referral thresholds. Participants thought that it usefully enabled different types of referrals for CYP, including referral with parents, with other family members, or in their own right:

There is very little support for children who have been living with parents when there’s been domestic abuse […] for them to have their own worker, someone they can do some work with […] it’s really beneficial for the children. (SM1).

Children are now recognised as victims of domestic abuse for the first time through the Domestic Abuse Act [26]. The increased recognition of harm strengthens the case for prevention and effective interventions to support CYP affected by DVA directly or indirectly [27]. Current service provisions are, however, according to one of the interview participants working directly with children, ‘*just focusing on the mum*’:

Children are now recognised as survivors in their own right […] but they don't have any funding in their own right. So they’re just an add-on. (CYPW2).

Given the pressures services for CYP are under, resulting in high referral thresholds and long waiting lists, clinicians valued the availability of a low threshold direct referral pathway to the IRIS + service, enabling early intervention for affected CYP:

*I’m really glad to have IRIS*+ *training […] so if we can pick up the domestic violence earlier and provide support earlier.* (GP4)

These thoughts were echoed by an IRIS + support service manager (SM1) who noted that, ‘*we do need to do the work with the children now, not later on in life*.’

Professionals delivering the IRIS + support for CYP felt that the ‘*one point of entry’* for all family members affected by DVA opened opportunities of support for CYP that otherwise would have been missed:

[P]articularly around mental health or emotional difficulties, it can sometimes feel like there isn’t anywhere to go with it. (CYPW1).

Reflecting on his experience of being referred to IRIS + by his GP, a young person who sought help for anger issues spoke about the unavailability of support for young people who have relationship difficulties.

‘The doctor that I saw, he was new and he said there’s hardly anything he can do because I’m neither an adult or a child […] but he’ll try and look for someone to help me with my anger and he found [name of IRIS + hub], and I’ve been doing it ever since. (CYP4, age 16).

#### Overcoming barriers to identifying CYP in their own right

Professionals working with CYP recognised that, although many children received IRIS + support in their own right around DVA, entrenched barriers to identifying CYP via primary care were difficult to overcome. This resulted in missed opportunities for supporting more CYP directly. As a social worker IRIS + trainer explained:

I think there are real fears of mothers, particularly, about being referred to professional services and that’ll act as a huge barrier, an understandable barrier, to bring in social services. (SW1).

According to clinicians and IRIS + support workers, fear of professional intervention is common among mothers seeking help for their children through primary care. Many might be concerned that an intervention around DVA for them or for their children might result in their children being removed from their care. A CYPW addressed these fears early on in the support process to reassure parents:

If we were social services, for example, then I think it might raise their anxiety a little bit in thinking that they're not doing something right. I think usually because we're a charity and we’re domestic abuse services and, you know, we kind of explain all of that when I phone and I think they're usually willing to engage. (CYPW2).

Another frequently discussed barrier to overcome during the process of direct DVA referral for CYP included the limited opportunities for detection in the primary health care setting, including *‘having that conversation with mum and kid together, and also the time factor’ –* said one of the IRIS + AEs (AE2). Another professional delivering work for CYP pointed out that, *‘children aren’t necessarily going to a doctor with the things that would maybe signpost to domestic abuse.’* She explained that due to time pressures, the *‘priority is getting that person [adult] the right help for their specific needs, and not necessarily thinking about everybody else within that family, unless there’s, like, a safeguarding issue.’* (CYPW1).

Identification of CYP affected by DVA was more common in face-to-face appointments built on pre-existing relationships with families. Although clinicians effectively used information received from third parties about CYP DVA exposure, they were concerned about the invisibility of CYP affected by DVA in remote consultations*. ‘We’re not seeing children, we’re not seeing whether they’re scruffy, unkempt, bruised’*—explained a GP, who felt that remote consultations compromised clinicians’ ability to recognise DVA in families:

All the cues that you would have got before, you’re not getting. It’s reliant on us remembering which of our patients had some issues, and we often have things like confidential data that flashes up, or a vulnerable child and family or something like that that makes me think, “Oh, I’ve got to be really a bit more aware.” […] It’s a different way of practising now, you’re not using your eyes. (GP8).

*‘You just can’t build that same therapeutic relationship with somebody over the phone’*, expressed a health visitor (HV1). An urgent care practitioner (UCP1) noted that in face-to-face consultations, they could *‘say to mum, "could you step out for a minute?", and I will just have a chat with the child’*. Remote consultations reduced opportunities to speak to children alone. *‘Lots of children tend to not want to speak over the telephone anyway and I end up speaking with their parents’*, she explained.

#### *Impact of IRIS* + *support on CYP*

Support received by CYP included regular (usually weekly) one-to-one support sessions offering emotional and practical support. It might have also included onward referral to specialist support including mental health support, play therapy, or group programme on healthy relationships. Parents, predominantly women, in addition to a range of specialised, trauma-informed needs-led one-to-one and practical support, also accessed emotional support and advice around parenting and childcare, referral to parent/child activity sessions or parenting courses, legal advice around child contact and resident arrangements, and support with access to safe accommodation.

The input she [mother] got from IRIS + was really good and quite comprehensive, because some of the children were also offered support and stuff which wasn’t what I had necessarily been expecting. […] I must admit, I was impressed with the level of input that was offered to this lady and her family. (GP7).

A young person described the activities as ‘fun’, and recalled how during a session with the CYPW, they: *‘did a piece of work that singles out what’s my triggers for these situations’.* He added that, *‘every time we do work, we have a joke and a laugh, and our other work, we have a lot of fun. So, it’s quite nice.’* (CYP4, age 16).

According to a CYPW, CYP who have received direct support ‘*seem more assertive, they seem more confident, […] they appear to be calmer, more in control of themselves.*’ In addition, parents supported by IRIS + *‘have really felt that they’ve learnt a lot from the support. They noticed things like the communication between them and their child, but also recognising improvement in their child as well’* (CYPW1). Reflecting on the interruptions to service delivery caused by the pandemic, an IRIS + support service manager (SM1) noted that although they have *‘achieved some good outcomes for the children’*, children* ‘would have maybe benefited from more face-to-face support in that ideal world.’*

In line with our previously reported findings on the benefits of IRIS + support on CYP [18], the direct and indirect support improved family relationships and led to improved mental health, wellbeing and confidence for CYP. *‘It makes me calmer. It makes me feel like I can just talk to her [CYPW] about anything, really* […] *She's helped a lot with it’*, said a child who received dedicated one-to-one support for six months (CYP3, age 12). Another child noted that following the sessions with the CYPW, he becomes* ‘a little bit less annoying’* for his brothers. (CYP2, age 11).

CYP voiced their appreciation for the support they had been given. As one young person summed up his experience with the intervention:

I’m a lot happier now. I’m coping. Even my family said that the work I’m doing is really, really helping me. […] I used to have, like, a really heavy load on myself. […] I’ve now begun coming out of my bedroom and started talking to my mum more, and started to leave the house more now, and starting to make friends again. Yes, so it’s been a pretty good thing. (CYP4, age 16).

## Discussion

### Summary: identifications and referrals

We tested the acceptability and feasibility of IRIS + , an adapted multi-sectoral IRIS programme. The IRIS + intervention tested in this study was based on evidence from our previous study [19] which has informed the reconfiguration of the intervention to better respond to the diverse needs of adult (women and men) and child patients living with DVA and/or experiencing it first-hand.

We found that the intervention led to improvements in clinical practice. Completion of clinical training and working within the IRIS + referral and support structure improved clinicians’ self-reported preparedness to respond to the needs of all patient groups, including female and male survivors, perpetrators, CYP and their parents. Consistent with previously reported findings [18, 19], the IRIS + training and support programme was highly valued by clinicians, service provider professionals and patients participating in the study. The popularity of the intervention translated to good clinician and patient engagement with IRIS + and to high rates of referrals for all patient groups, including men (mostly survivors) and CYP. The identification of patients through external (third party) reports about DVA incidents facilitated the referral work, particularly through the pandemic period, which saw a one third reduction in all IRIS + referrals.

During intervention development, a potential unintended consequence considered was that engagement with men and CYP in IRIS + could lead to a reduction in referrals of women DVA survivors. However, conversely, the added intervention components on men and CYP increased the referral rate for women. Comparing referral numbers in IRIS + to the original IRIS Programme, while IRIS + also received referrals for men (10% of all referrals) and direct referrals for CYP (15% of all referrals), the referral rate for women was more than double than that of the original IRIS trial [11]. In addition to direct CYP referrals, there were a very large number of CYP identified and referred together with their parents due to potential DVA exposure. Over two-thirds of referred women and CYP and almost half of all referred men (all survivors) were directly supported by the IRIS + service. The small number of men perpetrators (2% of all referrals) were offered referral to perpetrator group programmes. Many CYP also received IRIS + support indirectly, via the referred parents.

### Comparison with existing literature

A pre-existing relationship between the clinician and the patient, and the face-to-face consultation were seen by both patients and clinicians as key enablers of DVA disclosure. Our study extends previous findings about continuity of care as a key component of effective DVA management and support in primary care [28, 29].

The study also widens our current understanding about the value and dynamics of collaboration within the primary care team in the context of DVA care [30–32]. The wide inclusion of clinicians affiliated with local primary care teams enabled the identification and referral of women, men and child patients using a collaborative whole team approach. This extended to collaboration between the primary care team and the IRIS + service support team. Ongoing communication between clinicians supporting affected patients and the link with named AEs contributed to safe DVA care. Effective whole team working, as found by Dixon et al. [32], and the proactive use of external DVA information helped to mitigate reduced opportunities for disclosure of DVA caused by the erosion of continuity of care and the shift to remote care. Primary care teams, by training together and sharing information, generated a high number of referrals across all patient groups. Most referrals, however, still came from GPs, indicating possibly missed opportunities for direct referrals from a wider range of primary care clinicians.

Men survivors supported by IRIS + spoke positively about their experiences of disclosure and referral. Consistent with previous research about both the initial and longer-term benefits of the IRIS style referral in relation to women [33], men participating in the study also reported positive impact of support, including improved physical and mental health, wellbeing and confidence.

Our interviews with clinicians and men survivors contribute to our understanding of common barriers which are difficult to overcome during the process of DVA disclosure. The most frequently discussed barriers that reduced opportunities for disclosure and identification for men in our study included the weakening of continuity of care and strong societal perceptions about masculinity. Our study confirms previously identified barriers to men seeking help [34] and to clinicians providing support for men affected by DVA [19, 22, 35, 36]. Structural barriers to men DVA disclosure and uncertainty about how to phrase questions to men about potential abusive behaviour during consultations (despite clinicians’ increased self-reported preparedness to respond to this patient group) were reflected in the small number of referrals for men perpetrators. These findings are in line with previous work where increased primary care clinicians’ confidence to identify and respond to men perpetrators and survivors did not lead to actual patient referrals [22]. They also echo findings on referral pathways to domestic violence perpetrator programmes, noting very few referrals from GPs or mental health services [37].

Clinicians and service providers thought that IRIS + had filled a service gap and was a particularly valuable resource in identifying CYP who fell below child protection service referral thresholds. In line with previous evidence [11, 38], CYP valued having their experiences validated and being listened to in the context of a trusting relationship with professionals. CYP receiving IRIS + support from the CYPW reported improved mental health outcomes and improved confidence. Clinicians were concerned about the invisibility of CYP affected by DVA in remote consultations. Structural barriers to direct identification of CYP via primary care were difficult to overcome, particularly in the pandemic period. This resulted in missed opportunities for supporting more CYP directly.

### Strengths and limitations

A key strength of our study is the multi‐agency and multi-professional collaborative approach taken during the intervention reconfiguration, delivery, and feasibility work. Another strength relates to the active involvement of two service user expert groups with lived experience of DVA. The study also benefited from including a variety of participant perspectives, including those of primary care clinicians in diverse roles, as well as the perspectives of diverse groups of patients, including the voices of CYP.

We explored aspects of feasibility throughout the whole care journey from seeking help through primary care to receiving specialist DVA support. The combination and comparison of quantitative and qualitative data to explore dimensions of feasibility and acceptability helped to strengthen the interpretation of findings [39].

As the study was testing the feasibility and acceptability of the intervention, it included only a small number of general practices. We tried to ensure the diversity of study practices in terms of size, location, and population, as well as the diversity of research participants. EMR and PIM + data interpretations were restricted by the binary nature of the medical record (female/male).

Another limitation is potential participation bias: the views of clinicians and patients participating in the study might reflect the perspectives of those individuals who may have had specific interests or expertise in DVA care or may have been more favourably disposed to the intervention. Moreover, the lack of men perpetrator participants limited the interpretation of findings. Although the study articulated some of the barriers that might prevent survivors of DVA and other family members disclosing DVA in general practice, the study did not explore why some people experiencing or perpetrating DVA do not seek or accept professional support. Further research is required to explore the perspectives of unidentified and/or unsupported primary care patients affected by DVA.

The study started before the emergence of the COVID-19 and covered a period of disruption caused by the pandemic. The pandemic led to important changes in working practices within primary care and changes to patient access. Data collection took place in a period of unprecedented pressures on primary care and extreme uncertainty for patients affected by DVA. Adaptations to data collection focus and methods were required, and lower follow-up response rates among both clinicians and patients were inevitable.

### Implications for feasibility

The IRIS + training and support intervention was acceptable to clinicians, service providers and patients, and was feasible to implement in English and Welsh urban areas in both IRIS-trained and non-IRIS trained general practices. The study also highlights the feasibility of research engagement with and data collection from general practice, DVA agencies and a vulnerable patient population of women and men survivors and CYP within the IRIS + intervention setting.

The study shows that the intervention extended the healthcare response beyond women survivors of DVA to the identification and referral of men and the *direct* identification and referral of CYP. Confirming the steps of change outlined in our logic model (Fig. 2.), our findings indicated changes in short-term outcomes for clinicians and patients, including (i) *increase in clinician’s confidence and preparedness to identify and respond to women, men and CYP affected by DVA*; (ii) *clinicians’ feeling of being supported in delivering DVA work for all patients;* (iii) *increase in DVA enquiry, disclosure, referral for women, men and CYP*; (iv) *enhanced recording of DVA*; and *women, men and CYP access specialist support*. (Fig. 2). Our findings on the strengthened clinician and patient engagement in relation to the wide range of short-term outcome domains provide strong evidence for the feasibility of the intervention to respond to the needs of women and men survivors and CYP living with DVA and/or experiencing it first-hand. The low number of men perpetrator referrals also suggests that previously reported barriers to referring perpetrators from primary care to specialist DVA support [19, 22, 35] proved to be difficult to surmount during the study period, despite increased preparedness and confidence reported by clinicians after training in this complex area of practice.

Although the testing of medium and long-term patient outcomes was outside the scope of the feasibility study, interviews with women, men and child patients supported by IRIS + , indicated (i) *improved mental and physical health outcomes*; (ii) *increased safety outcomes*; (iii) *improved quality of life*; and *strengthened multi-sectoral prevention response to DVA.* Given that it was beyond the aims of the current study to examine longer term implementation, there remains uncertainty about the scalability and sustainability of the adapted intervention (Fig. 2).

## Conclusion: implications for research and practice

Our testing of the reconfigured IRIS + intervention has demonstrated acceptability and feasibility for women and men survivors and CYP. This study did not test effectiveness or actual cost-effectiveness, and whether, if implemented on a larger scale, it would reach a wide range of professionals and patients, particularly men who are using violence. More research is needed to support effective approaches to identification of men perpetrators within the health setting. Future research should also explore reasons for the increase in women referrals in the context of whole-family DVA interventions.

Building on current evidence of feasibility, the next step should be to fully evaluate the implementation scalability, effectiveness, cost-effectiveness and impact of IRIS + in different contexts to ensure generalisability. Rigorous testing of IRIS + will provide key evidence about benefits through targeting secondary prevention and reduced healthcare service use. Improved identification, referral and health outcomes, and downstream benefit for survivors and CYP demonstrated through cost-effectiveness modelling would form a strong basis for service commissioning and hence sustainability. It would also inform policy and practice by generating evidence about the extent to which local variations in implementation contexts facilitate or impede intervention effectiveness and reach.

### Supplementary Information

**Additional file 1. **

**Additional file 2. **

**Additional file 3. **

**Additional file 4. **

**Additional file 5. **

**Additional file 6. **

**Additional file 7. **

**Additional file 8. **

**Additional file 9. **

**Additional file 10. **

**Additional file 11. **

## Data Availability

Anonymised transcript and questionnaire data will be stored on the University of Bristol’s Research Data Service Facility. Bona fide researchers will be able to access non-identifiable data upon reasonable request. Given the highly sensitive nature of DVA research, we will adopt trauma-informed data sharing approaches, still consistent with open science. Access will be subject to a data access agreement and following approval from the REPROVIDE Chief Investigator (Professor Gene Feder, gene.feder@bristol.ac.uk) and the University of Bristol Data Access Committee.

## Abbreviations

AE: Advocate educator
CL: Clinical lead
CYP: Children/young people
CYPW: Children and young persons’ worker
DVA: Domestic violence and abuse
EMR: Electronic medical record
GP: General practitioner
HCA: Health care assistant
HV: Health visitor
IRIS: Identification and Referral to Improve Safety
IRIS +: Enhanced Identification and Referral to Improve Safety
MARAC: Multi-agency risk assessment conference
PIM: PROVIDE Intervention Measure
PN: Practice nurse
REPROVIDE: Reaching Everyone Programme of Research On Violence in diverse Domestic Environments
SALW: Substance abuse liaison worker
SM: Service manager
SW: Social worker
UCP: Urgent care practitioner
