# Supplementary material for: Feasibility of a reconfigured domestic violence and abuse training and support intervention responding to affected women, men, children and young people through primary care
Source: BMC Prim Care. 2024 Jan 26;25:38. doi: 10.1186/s12875-023-02249-5 (PMC10811857; doi:10.1186/s12875-023-02249-5)
Supplement: Supplementary file 2 — Additional file 2. [file 12875_2023_2249_MOESM2_ESM.pdf]

Follow up clinician IRIS+ survey

IRIS+ study to test the feasibility of a training and support intervention for general practice to improve the response to women, men and children exposed to domestic violence and abuse

Thank you for agreeing to complete this brief follow-up survey.

Your participation in this **survey** will help us to **evaluate and improve** the **IRIS+ domestic violence training and support intervention.**

The aim of this follow-up questionnaire is to **assess the impact of the IRIS+** to your daily practice. We are assessing the extent to which the learning form the **IRIS+ training has been put into practice** since your training. We are also interested to know to what extent the training and support intervention increased your **confidence and preparedness** in relation to the management of domestic violence and abuse cases.

Remember, we are assessing the effects of the training intervention – not you!

We are interested in the average learning and behavioural impact of the training sessions; we are not concerned with individual answers and individual results will be strictly confidential.

Any information you give us will be treated as **confidential**. We will not pass on any identifiable information in relation to your responses to your surgery, the trainers or anyone else. We will store information collected in the study in locked filing cabinets and on password protected databases. You can choose to not participate at any time.

Please fill in your answers based on how confident and prepared you feel today.

Please answer as honestly as you can.

Please check the box below to confirm your consent to taking part in this study.

☐ I consent to taking part in this research.

Section 1. Confidence and preparedness

This section asks how confident and prepared you feel now (after completing the IRIS+ training) to perform various tasks in relation to the management of patients presenting with issues that may be related to domestic violence and abuse.

All information will be treated in confidence and anonymised.

Throughout this survey, the term domestic violence and abuse or domestic violence is used. This refers to: "any incident or pattern of incidents of controlling, coercive, threatening behaviour, violence or abuse between those aged 16 or over who are, or have been, intimate partners or family members regardless of gender or sexuality. The abuse can encompass, but is not limited to psychological, physical, sexual, financial, emotional." (Cross-government definition of domestic violence and abuse, 2016)

Section 1.

Please indicate how prepared you feel to perform the following tasks for the different patient groups.

Ask appropriate questions about domestic violence and abuse to:

	Not prepared	Slightly prepared	Moderately prepared	Fairly well prepared	Well prepared
Females who may be victims	○	○	○	○	○
Females who may be perpetrators	○	○	○	○	○
Males who may be victims	○	○	○	○	○
Males who may be perpetrators	○	○	○	○	○
Parents about their children's exposure to domestic violence	○	○	○	○	○
Children or young people	○	○	○	○	○

Identify signs and symptoms associated with domestic violence and abuse for:

	Not prepared	Slightly prepared	Moderately prepared	Fairly well prepared	Well prepared
Females who may be victims	○	○	○	○	○
Females who may be perpetrators	○	○	○	○	○
Males who may be victims	○	○	○	○	○
Males who may be perpetrators	○	○	○	○	○
Parents about their children's exposure to domestic violence	○	○	○	○	○
Children and young people	○	○	○	○	○

Provide appropriate initial response to disclosures about domestic violence and abuse from:

	Not prepared	Slightly prepared	Moderately prepared	Fairly well prepared	Well prepared
Females who may be victims	○	○	○	○	○
Females who may be perpetrators	○	○	○	○	○
Males who may be victims	○	○	○	○	○
Males who may be perpetrators	○	○	○	○	○
Parents about their children's exposure to domestic violence	○	○	○	○	○
Children and young people	○	○	○	○	○

Make appropriate referrals for:

	Not prepared	Slightly prepared	Moderately prepared	Fairly well prepared	Well prepared
Females who may be victims	○	○	○	○	○
Females who may be perpetrators	○	○	○	○	○
Males who may be victims	○	○	○	○	○
Males who may be perpetrators	○	○	○	○	○
Parents about their children's exposure to domestic violence	○	○	○	○	○
Children and young people	○	○	○	○	○

Safely and appropriately record disclosure and suspicion of domestic violence and abuse in medical records for:

	Not prepared	Slightly prepared	Moderately prepared	Fairly well prepared	Well prepared
Females who may be victims	○	○	○	○	○
Females who may be perpetrators	○	○	○	○	○
Males who may be victims	○	○	○	○	○
Males who may be perpetrators	○	○	○	○	○
Parents about their children's exposure to domestic violence	○	○	○	○	○
Children and young people	○	○	○	○	○

Provide ongoing support in my clinical role for:

	Not prepared	Slightly prepared	Moderately prepared	Fairly well prepared	Well prepared
Females who may be victims	○	○	○	○	○
Females who may be perpetrators	○	○	○	○	○
Males who may be victims	○	○	○	○	○
Males who may be perpetrators	○	○	○	○	○
Parents who have children exposed to domestic violence	○	○	○	○	○
Children and young people	○	○	○	○	○

You have now nearly completed the survey. Please answer a final question regarding prevalence.

How many new diagnoses of domestic violence and abuse have you made since mid-May 2017 (your first IRIS+ training session) for the following patient groups:

	None	1-2	3-5	6-10	11-20	More than 20	N/A
Females who may be victims	○	○	○	○	○	○	○
Females who may be perpetrators	○	○	○	○	○	○	○
Males who may be victims	○	○	○	○	○	○	○
Males who may be perpetrators	○	○	○	○	○	○	○
Parents who have children exposed to domestic violence	○	○	○	○	○	○	○
Children and young people	○	○	○	○	○	○	○

If you have any additional comments, concerns or questions either in relation to the IRIS+ training or this survey, please use the text box below.
You can also email your comments to the research team on iris-plus@bristol.ac.uk
