# Supplementary material for: Feasibility of a reconfigured domestic violence and abuse training and support intervention responding to affected women, men, children and young people through primary care
Source: BMC Prim Care. 2024 Jan 26;25:38. doi: 10.1186/s12875-023-02249-5 (PMC10811857; doi:10.1186/s12875-023-02249-5)
Supplement: Supplementary file 3 — Additional file 3. [file 12875_2023_2249_MOESM3_ESM.pdf]

Study to test the feasibility of a training and support intervention for general practice to improve the response to women, men and children exposed to domestic violence and abuse (DVA) – Stage 2

Interview Schedule for primary healthcare professionals – Time point 1*

Introductory statement

Thank you for agreeing to do this interview. I would like to ask you some questions about how you felt about the domestic violence training that you recently attended and how you feel it has impacted on your clinical practice. The interview will last between 20-30 minutes. If there are any questions that you don't feel comfortable answering, just tell me and I'll move on to another topic. Or, if you decide you want to stop the interview altogether that's fine, just let me know. Our conversation today is completely confidential.

We are interested in hearing about your views and experiences. Although I have a list of questions, please do mention anything that you think is important that I don't ask you. If anything is unclear during the interview, let me know.

Consent checklist

Check participant:

- Has read the participant information sheet
- Understands that their participation is voluntary and that they can change their mind and withdraw at any time without having to give a reason and this won't impact on the support that they are receiving from the IRIS+ service.
- Understands that if I have serious concerns about their safety, or that of any children they mention, that I may need to share this concern with an appropriate agency.
- Understands that personal information about them (such as my name and address) will be treated with strict confidence and securely stored separately from all other data about them (e.g. interview transcripts) at the University of Bristol.
- Agrees that the anonymised information collected about them (anonymised transcripts) may be used to support the current research and relevant future research and may be shared anonymously.

Any questions?

Consent for recording

With your permission, I'd like to digitally record the interview. This is so the interview can be transcribed. It will be erased after being transcribed. All names of people or places which might identify you or others will not be transcribed. Are you happy to continue with the interview and for it to be digitally recorded?

The recording of the interview will be kept securely and only the anonymised transcript of that interview will be used within the research. The recordings themselves will be erased after transcription.

Can I just confirm that I have your consent to be audio-recorded while this interview takes place?

I'll turn the recorder on now then, and for the record state:

Today's date is…… my name is……… and your name is ……… and I have your consent to carry out/record this interview? (yes)

Introductory question

Check RedCap PIM+ answers prior to interview and if necessary, follow-up with this introductory question as follows:

It would be helpful if you briefly describe your usual day-to-day role at [practice name] in regard to patient contact and relevant responsibilities regarding our DVA client group.

→ **Prompts to be used if needed:**

- Explore any relevant activities where they may come into contact with adults/children affected by DVA.

Can you also describe the changes to your practice during the COVID-19 lockdown measures?

→ **Prompts to be used if needed:**

- Patient contact
- Timing of changes
- Impact on themselves
- Impact on patients
- Main barriers to practice as usual
- Any facilitators/positives from this?
-

Training reflections

Check/confirm with participant how many sessions they attended/were provided to their practice.

*If no IRIS+ training go to last question in this section**

If participated in IRIS+ training

Can you tell me what you remember about the first training session?

→ **Prompts to be used if needed:**

- Can you talk me through what you did?
- What the focus of the session was on?
- Take home message? / Most useful thing you gained from the training?

IF APPROPRIATE: Can you tell me what you remember about the second training session?

→ **Prompts to be used if needed:**

- Can you talk me through what you did?
- What the focus of the session was on?
- Take home message? / Most useful thing you gained from the training?

Which parts/aspects of the training did you think were the most useful for your professional role? Did you learn any new skills from the training?

→ **Prompts to be used if needed:**
- Why was it useful?
- What has this training helped you do in practice?
- Were there any aspects that you felt were not relevant to your role? If yes, how did you feel about it's inclusion?

What aspects of the training were not useful or helpful?

→ **Prompts to be used if needed:**
- Can you describe what didn't work so well in the training?
- Was material repetitive?
- Already known to audience?
- Not explained enough?

What is your opinion on the style and format of the sessions?

→ **Prompts to be used if needed:**
- What do you think we could do to improve the training?
- Did you use the online resources?
- Quality of trainers – e.g. substantive knowledge/presentation/ability to answer questions
- Length of training
- Can you think of anything that would make it better for the future?
- How could we make it more relevant to your role?
- Any evidence it has helped the team e.g. discussions in practice meetings?

What was missing from the training that you would have liked to be included and why?

Any IRIS+ Covid-19 online refresher training attended?

→ **Prompts to be used if needed:**
- Barriers/facilitators to attending online training?
- Can you talk me through what you did?
- What the focus of the session was on?
- Take home message? / Most useful thing you gained from the training?

**If they have not participated in any IRIS+ training*

Have you had any previous DVA training?

→ **Prompts to be used if needed:**
- Timing of this?
- How would you assess your knowledge of DVA and local support available for patients?
- How best could they be included in future IRIS+ training?
- Location ect?

Training for non-GP primary care staff
**How do you think the training could be improved for non-GP primary care staff?**<br><br>→ **Prompts to be used if needed:**<br>- Content/relevance<br>- Location of training<br>- Timing of training<br>- Style of training<br>- Barriers and facilitators to training

Implementation of training
If participated in IRIS+ training<br><br>*Begin with personalisation i.e. summary of referrals from [practice name]*<br><br>**We know that some training is quite easy to go away and implement immediately and that other training is harder to put into application. How have you found it with the IRIS+ training?**<br>→ **Prompts to be used if needed:**<br>- What have you done differently in your practice since the training?<br>- Has your opinion changed in any way? E.g. in relation to men being victims/perpetrators, women being victims/perpetrators, children being exposed to DVA?<br><br>*All participants*<br>**Have you responded to a situation where you suspected a female or male patient was a victim or perpetrator of domestic violence - can you give me some examples?** [Refer to a specific case if known]<br>→ **Prompts to be used if needed:**<br>- How did you feel asking patient about DVA?<br>- Did it impact on relationship with patient?<br>- Did you make a referral? Why/why not? (may want to probe if men willing for a referral to happen)<br>- Have any difficulties arisen as a result of your enquiries re DVA?<br>- Did the/any training prepare you for these situations? Why/Why not?<br>- Any other thoughts about this?<br><br>**Have you responded to a situation where you suspected a child patient was exposed to DVA – can you give me some examples?**<br>→ **Prompts to be used if needed:**<br>- How did you feel asking child or parent/carer about DVA?<br>- Did it impact on relationship with patient either parent or child?<br>- Did you make a referral? Why/why not? (may want to probe if parent/carer/child willing for a referral)<br>- Have any difficulties arisen as a result of your enquiries re DVA?<br>- Did the training prepare you for these situations? Why/Why not?<br>- Any other thoughts about this?

Have you made any referrals to Children's Social Care because you were concerned about a child safeguarding issues? If yes

→ **Prompts to be used if needed:**

- Did you discuss the matter with another professional before making a referral?
- Did the training support your identification of potential safeguarding issues in relation to DVA? Why/why not?
- Do you know what the outcome of the referral was?
- Any other thoughts about the referral process?

Were there any obstacles to referring to the IRIS+ DVA service?

→ **Prompts to be used if needed:**

- What were these?
- Could the researchers or advocate educators done anything to help make things easier?
- Did patients refuse to be referred?
- Can you tell us why they refused?

Are there specific barriers and facilitators to referral for non-GP primary care staff? What are these?

→ **Prompts to be used if needed:**

- Role and responsibility
- Knowledge and experience
- Capability and opportunity
- Pragmatic – access to medical notes
- Norms and hierarchy

Impact of COVID-19 on practice and IRIS+ intervention

How have general practices adapted to social distancing measures?

→ **Prompts to be used if needed:**

- Remote consultations – effectiveness, sensitive issues such as DVA
- Changes to presenting problems of patients.

Impact of COVID-19 lockdown measures on the IRIS+ intervention

→ **Prompts to be used if needed:**

- Changes to how you respond to potential DVA?
- Facilitating disclosure?
- Does this vary by the gender/age of the patient?
- Has this varied as time has gone or as lockdown measures have eased?
- Feasibility of delivering IRIS+ intervention remotely?
- Challenges/facilitators to offering referral and support to DVA service

3rd party referrals and working with others

Have you received any 3rd party referrals since your training?

- If so, can you talk me through what you did with these referrals/information?
- Did your attendance at the IRIS+ training influence the actions you took/plan to take (now or in the future)?

Support

What support (either formal or informal) have you been provided with since the training?(e.g. links with advocate educator, social worker)

→ **Prompts to be used if needed:**

- How useful has it been?
- Is it enough support or is more needed?
- What kind of support do you think health professionals need in order to deal with domestic violence in their clinical practice?
- Anything else that could be useful? E.g. email reminders, support from AE?

Future training

Would you like any further training on domestic violence – what aspects would you be most interested in and why? E.g. further training needs

Conclusion

Let the participant know that it's the end of your questions and ask them if there are any other comments that they would like to make.

Thank participant for their time.

*Please note this is a suggested guide for interviews only. In keeping with standard practice in qualitative research, these questions may be modified & added to as the trial progresses and new themes or areas of interest emerge. We will also adapt this guide for use with participants at earlier or later stages of the study.
