# Supplementary material for: Feasibility of a reconfigured domestic violence and abuse training and support intervention responding to affected women, men, children and young people through primary care
Source: BMC Prim Care. 2024 Jan 26;25:38. doi: 10.1186/s12875-023-02249-5 (PMC10811857; doi:10.1186/s12875-023-02249-5)
Supplement: Supplementary file 4 — Additional file 4. [file 12875_2023_2249_MOESM4_ESM.pdf]

Study to test the feasibility of a training and support intervention for general practice to improve the response to women, men and children exposed to domestic violence and abuse (DVA) – Stage 2

Interview Schedule for primary healthcare professionals – Time point 2*

Introductory statement

Thank you for agreeing to do this interview. I would like to ask you some questions about how you feel the IRIS+ training has impacted on your clinical practice since we last spoke in [month]. The interview will last between 20-30 minutes. Like last time, if there are any questions that you don't feel comfortable answering, just tell me and I'll move on to another topic. Or, if you decide you want to stop the interview altogether that's fine, just let me know. Our conversation today is completely confidential.

We are interested in hearing about your views and experiences. Although I have a list of questions, please do mention anything that you think is important that I don't ask you. If anything is unclear during the interview, let me know.

Consent checklist

Check participant:

- Has read the participant information sheet
- Understands that their participation is voluntary and that they can change their mind and withdraw at any time without having to give a reason and this won't impact on the support that they are receiving from the IRIS+ service.
- Understands that if I have serious concerns about their safety, or that of any children they mention, that I may need to share this concern with an appropriate agency.
- Understands that personal information about them (such as my name and address) will be treated with strict confidence and securely stored separately from all other data about them (e.g. interview transcripts) at the University of Bristol.
- Agrees that the anonymised information collected about them (anonymised transcripts) may be used to support the current research and relevant future research, and may be shared anonymously.

Any questions?

Consent for recording

With your permission, I'd like to digitally record the interview. This is so the interview can be transcribed. It will be erased after being transcribed. All names of people or places which might identify you or others will not be transcribed. Are you happy to continue with the interview and for it to be digitally recorded?

The recording of the interview will be kept securely and only the anonymised transcript of that interview will be used within the research. The recordings themselves will be erased after transcription.

Can I just confirm that I have your consent to be audio-recorded while this interview takes place?

I'll turn the recorder on now then, and for the record state:

Today's date is…… my name is……… and your name is ……… and I have your consent to carry out/record this interview? (yes)

Interviewer provides participant with update on referrals made to IRIS+ service and the demographics of these referrals.

Experiences of using the referral process and implementation of training into practice

How have you found the referral process (if you have used it)?

→ **Prompts to be used if a referral made:**

- Describe referral process for your practice (as may differ)
- How could this be improved?
- Anything that is a barrier to you referring?

→ **Prompts to be used if no referral made:**

- What are the reasons you have not referred anyone to the DVA service?
- Is there anything you need to in order to make referrals? (e.g. refresher training, time ect)

Has the referral process changed during the COVID-19 lockdown and ongoing pandemic restrictions, and how?

→ **Prompts to be used if needed:**

- Patient contact
- Timing of changes
- Impact on themselves
- Impact on patients
- Main barriers to practice as usual
- Any facilitators/positives from this?

What has been your experience of implementing IRIS+ training in your day-to-day clinical practice? (e.g. asking men, women and children about potential DVA)

→ **Prompts to be used if needed:**

- Positive and negative experiences of asking?
- Own sense of competence/confidence to ask/deal with DVA?
- Awareness of wider issues impacting on health for your patients such as DVA?

Have you experienced any barriers or challenges in asking patients about DVA or referring those patients to IRIS+? Please describe.

Would you feel comfortable asking a male patient about domestic violence and abuse? If you would not, or would feel unsure about doing so, what are the reasons for this? Has this changed since you attended the IRIS+ training?

Would you feel comfortable asking a child or young person about domestic violence and abuse? If you would not, or would feel unsure about doing so, what are the reasons for this? Has this changed since you attended the IRIS+ training?

Thinking about your surgery/the surgery you are affiliated with, what are your colleagues' experiences of implementing IRIS+?

Do you have any suggestions as to how we can improve the IRIS+ training to better prepare primary care staff and increase the number of identifications of domestic violence and abuse and referrals to our specialist service (particularly men and children)?

Do you have any suggestions as to how we can improve the IRIS+ referral pathway to increase the number of identifications of domestic violence and abuse and referrals to our specialist service (particularly men and children)?

Have you had a chance to look at the new online resource? If so, what are your impressions of it? What is helpful/not helpful?

Have you received any 3rd party reports in relation to DVA since attending the training? If so, please talk me through how the report(s) were processed and if attending the IRIS+ training impacted on this.

IRIS+ referrals and non-GP primary care staff

In general, what are your thoughts about non-GP healthcare professionals referring patients via the IRIS+ service?

→ **Prompts to be used if needed:**

- Barriers to referral
- Facilitators to referral
- What would need to change in order for non-medical health professionals to refer patients to the DVA service? [Training, intervention, organisational culture]

Conclusion

Let the participant know that it's the end of your questions and ask them if there are any other comments that they would like to make.

Thank participant for their time.

*Please note this is a suggested guide for interviews only. In keeping with standard practice in qualitative research, these questions may be modified & added to as the trial progresses and new themes or areas of interest emerge. We will also adapt this guide for use with participants at earlier or later stages of the study.
