# Supplementary material for: Feasibility of a reconfigured domestic violence and abuse training and support intervention responding to affected women, men, children and young people through primary care
Source: BMC Prim Care. 2024 Jan 26;25:38. doi: 10.1186/s12875-023-02249-5 (PMC10811857; doi:10.1186/s12875-023-02249-5)
Supplement: Supplementary file 6 — Additional file 6. [file 12875_2023_2249_MOESM6_ESM.pdf]

Study to test the feasibility of a training and support intervention for general practice to improve the response to women, men and children exposed to domestic violence and abuse (DVA) – Stage 2

Interview Schedule for IRIS+ child support workers *

Introductory statement

Thank you for agreeing to do this interview. Introduce self. Today I would like to ask you some questions about your role as an IRIS+ support worker and how your service was received by children and young people. The interview will last between 20-60 minutes. If there are any questions that you don't feel comfortable answering, just tell me and I'll move on to another topic. Or, if you decide you want to stop the interview altogether that's fine, just let me know. Our conversation today is completely confidential.

I am interested in hearing about your views and experiences of the IRIS+ intervention in relation to your role. Although I have a list of questions, please do mention anything that you think is important that I don't ask you. If anything is unclear during the interview, let me know.

Consent checklist

Check participant:

- Has read the participant information sheet
- Understands that their participation is voluntary and that they can change their mind and withdraw at any time without having to give a reason and this won't impact on the support that they are receiving from the IRIS+ service.
- Understands that if I have serious concerns about their safety, or that of any children they mention, that I may need to share this concern with an appropriate agency.
- Understands that personal information about them (such as my name and address) will be treated with strict confidence and securely stored separately from all other data about them (e.g. interview transcripts) at the University of Bristol.
- Agrees that the anonymised information collected about them (anonymised transcripts) may be used to support the current research and relevant future research, and may be shared anonymously.

Any questions?

Consent for recording

With your permission, I'd like to digitally record the interview. This is so the interview can be transcribed. It will be erased after being transcribed. All names of people or places which might identify you or others will not be transcribed. Are you happy to continue with the interview and for it to be digitally recorded?

The recording of the interview will be kept securely and only the anonymised transcript of that interview will be used within the research. The recordings themselves will be erased after transcription.

Can I just confirm that I have your consent to be audio-recorded while this interview takes place?

I'll turn the recorder on now then, and for the record state:

Today's date is…… my name is……… and your name is ……… and I have your consent to record this interview? (yes)

Introductions

Briefly describe your current professional role?

→ **Prompts to be used if needed:**

- How often do you work with CYP who experience or are affected by DVA?
- What kinds of work do you do with CYP experiencing DVA?

Reflecting on your experience over the past 18 months, and as we near the end of the IRIS+ intervention, can you tell me about your experience and the thoughts and views that you have about it (the IRIS+ intervention)?

→ **Prompts to be used if needed:**

- Implementation
- Uptake by [name of agency]
- Uptake by general practices

Referral and first contact

Over the course of the IRIS+ intervention have the numbers and types of referrals been what you expected? Did anything about them surprise you? Have they changed over time?

Thinking about the referral process from the GP's/HCPS to your service…

What worked well and what would you like to improve?

→ **Prompts to be used if needed:**

- Referrals process, forms and appropriateness?
- Information from GPs/HCPs adequate?
- Children referrals – barriers/facilitators?
- Direct versus indirect referrals of children?
- How do you feel the referral of children has gone?
- How would you improve this aspect of the intervention – referral and support of children?

Has the referral process to IRIS+ changed during the COVID-19 lockdown and ongoing pandemic restrictions. If yes, how?

→ **Prompts to be used if needed:**

- Client contact
- Timing of changes
- Impact on themselves
- Impact on clients

- Main barriers to practice as usual
- Any facilitators/positives from this?

What other support would help GPs to engage with patients about DVA during the pandemic?

Were there variations between different practices (IRIS trained v naïve; size/location of practice) or professions of staff (GPs, other HCPs) and perceived reasons for this?

Were there variations between different practices (IRIS trained v naïve; size/location of practice) or professions of staff (GPs, other HCPs) and perceived reasons for this?

Thinking back to when you made contact with parents/carers about supporting their children/YP.

→ **Prompts to be used if needed:**

- How did the initial contact go? (e.g. was parent/carer expecting contact to be made, had they remembered that referral had been made)
- Did referred parent/carers express any concern or annoyance at being referred to your service?
- If parent/carer were not willing to meet with you/did not want their child to be supported what reasons did they give (if any)?

<u>Working with the general practice (if relevant)</u>

Can you tell me about how you have engaged and worked with the general practices and the individuals within them?

→ **Prompts to be used if needed:**

- Clinicians
- Safeguarding lead
- Communications – frequency and means (telephone, email, in-person)
- Visiting the practice – experiences of this

In general, how have you found interactions with GPs during the pandemic?

<u>Support work</u>

I want to turn to the kind of support that your service offers to children/YP, what that support looks like, how it was received by the children/YP and how you think it could be improved.

In your role as an Advocate Educator for the IRIS+ study, what support have you offered and provided for children/YPs?(You might like to consider specific and varied cases (e.g. those that benefited/didn't benefit, needs fulfilled/not fulfilled)**:**

- **Prompts to be used if needed:**
- Can you give some specific examples of support offered/engaged with?
- Did you feel prepared to work with children and YPs? If no, what would you need to become prepared?
- Did you feel supported by your organisation to work with children and YPs?

Do you think your service needs to develop and improve its support to children and young people? How could this be done?

Have IRIS+ children and young people needed or asked for support with things you could not help them with?

- If yes**,** can you give me some examples?
- What did you do in these situations (e.g. did you refer them on to other services etc)

How do you think the children/young people responded to the support offered by your service?

What impact (both positive and negative) do you think your support has had on the lives of children/young people you have worked with? Can you give some examples?

Impact of COVID-19 on practice and IRIS+ intervention

How has your professional practice adapted/changed in response to Covid-19 and the social distancing measures?

→ **Prompts to be used if needed:**

- Remote consultations – effectiveness, sensitive issues such as DVA
- Changes to presenting problems of patients, incidence of DVA.
- Changes to the types of support you can offer
- Has this varied as time has gone or as lockdown measures have varied?
- Challenges/facilitators to offering support to this client group
- Support given/needed to adapt to changes required
- Reorganisation of working practices
- Social distancing
- Changing relationships with colleagues
- Changing relationships with service users
- Reorganisation of work systems
- Staff sickness
- Boundaries between home and work

Looking forward

How do you think your support service needs to develop to meet the needs of children/YP being referred by primary healthcare professionals?

Having worked remotely during the pandemic, what are your thoughts and feelings about the feasibility of continuing remote working in the future?

→ **Prompts to be used if needed:**

- Possible losses/gains
- Types of work where it works / doesn't work?

- Is it being proposed within the organisation?

How do you think your support service needs to develop for IRIS+ to be rolled out in the future?

Conclusions

Let the participant know that it's the end of your questions and ask them if there are any other comments that they would like to make.

Thank participant for their time.

*Please note this is a suggested guide for interviews only. In keeping with standard practice in qualitative research, these questions may be modified & added to as the trial progresses and new themes or areas of interest emerge. We will also adapt this guide for use with participants at earlier or later stages of the study.
