# Supplementary material for: Feasibility of a reconfigured domestic violence and abuse training and support intervention responding to affected women, men, children and young people through primary care
Source: BMC Prim Care. 2024 Jan 26;25:38. doi: 10.1186/s12875-023-02249-5 (PMC10811857; doi:10.1186/s12875-023-02249-5)
Supplement: Supplementary file 7 — Additional file 7. [file 12875_2023_2249_MOESM7_ESM.pdf]

Study to test the feasibility of a training and support intervention for general practice to improve the response to women, men and children exposed to domestic violence and abuse (DVA) – Stage 2

Interview Schedule for IRIS+ trainers and AEs

Introductory statement

Thank you for agreeing to do this interview. Introduce self. Today I would like to ask you some questions about how you felt leading the domestic violence training for IRIS+ and how you feel it was received by your audience. The interview will last between 20-60 minutes. If there are any questions that you don't feel comfortable answering, just tell me and I'll move on to another topic. Or, if you decide you want to stop the interview altogether that's fine, just let me know. Our conversation today is completely confidential.

First of all it would be great to focus on your role as a trainer and your experiences of that - and then move on to think more specifically about your role as an AE

Consent checklist

Check participant:

- Has read the participant information sheet
- Understands that their participation is voluntary and that they can change their mind and withdraw at any time without having to give a reason.
- Understands that if I have serious concerns about their safety, or that of any children they mention, that I may need to share this concern with an appropriate agency.
- Understands that personal information about them (such as name and address) will be treated with strict confidence and securely stored separately from all other data about them (e.g. interview transcripts) at the University of Bristol.
- Agrees that the anonymised information collected about them (anonymised transcripts) may be used to support the current research and relevant future research, and may be shared anonymously.

Any questions?

Consent for recording

With your permission, I'd like to digitally record the interview. This is so the interview can be transcribed. All names of people or places which might identify you or others will not be transcribed. Are you happy to continue with the interview and for it to be digitally recorded?

The recording of the interview will be kept securely and only the anonymised transcript of that interview will be used within the research. The recordings themselves will be erased after transcription.

Can I just confirm that I have your consent to be audio-recorded while this interview takes place?

I'll turn the recorder on now then, and for the record state:

Today's date is…… my name is……… and your name is ……… and I have your consent to record this interview? (yes)

Introductions

Please can you describe your current professional role?

- How often do you work with women and men who are perpetrators of DVA and children who are exposed to DVA?

What motivated you to become an IRIS+ trainer?

Did you previously deliver the IRIS training? When?

Have you worked in similar or related roles before?

Will cover the responsiveness of the different practices to the training; if the format of the training worked for your audience and your thoughts on the content of the training.

Training delivery

Which training sessions did you deliver?

– What elements did you focus/lead on in these sessions?
– What preparation did you do in order to deliver the training?
– How comfortable and confident did you feel in talking about these topics? Did that change over time?

Who did you co-deliver the sessions with?

How well do you feel your co-delivery worked?
What worked well? Why/why not?

– **Do you think it was useful to have different professionals delivering the training?** Why/why not?

In general, what can you remember about the session at X? How responsive was the audience at X?

How responsive were your audience?

– Were different GPS/PHCPS practices more or less responsive? Can you think of any reasons for this? (which professionals were attending, group dynamics)
– Did the responsiveness of different groups of health professionals in your audience vary (e.g. drug and alcohol workers/Health Visitors) How? Why?
– Did they ask questions?
– Seek out information if they did not understand/were unclear?
– Raise any concerns or issues with the training?
– Did they work well together during the group and pair exercises?
– Answer questions when posed to the room?

Ask participant to reflect on the dynamics between different groups of Health professionals

How accepting were your audience(s) that identifying and responding to DVA was/should be part of their job? If they weren't at the beginning, do you think that changed over the course of the training?

From your perspective, did the format of the training work? What worked well/what didn't?

From your perspective, were there any particular exercises or delivery approaches that worked well or not so well? Why do you think this?
Prompts – video clips, group discussion, q and a, presenter delivering knowledge

Thinking about the delivery of the training:
What do you think could have been improved? E.g. timing, duration, location, who delivers the training, type of delivery (e-resource, face to face)

Ask participant to reflect on differences in experiences of delivering the IRIS+ training versus the IRIS training (if applicable).

<u>Training content</u>

Thinking about the content of the training:

– What role did you have in helping to develop the presentation slides or training material? Please describe.

What do you think worked well in relation to the training content? e.g. what pieces of information/knowledge appeared to be most useful to your audience

What do you think did not work well in relation to the training content? Were there pieces of knowledge and information which were known by the audience/not relevant to the audience?

Can you recall any content of the training which caused particular controversy or debate amongst the audience?
OR
Was there any content which required more explanation and attention than expected?
Prompts to explore this further:

– Why do you think the topic required extra attention/stimulated debate?
– Was it useful to be able to discuss the topic in more depth?
– Were you able to satisfy questions/debates about the content?

Do you think that the needs of the different groups of HCPs who attended the training sessions were addressed?

- Why/why not?
- What makes you think that?

What do you think was missing from the training content?

What do you think needs to be added to the training content in the future?

Overall evaluation (might leave out and save for follow-up interview)

The IRIS+ training aims to support GPs and other health professionals, including health visitors and drug and alcohol workers, to improve their responses to male and female victims and survivors of DVA and children exposed to DVA.

How successful do you feel the training was in supporting primary healthcare professionals to identify and support female survivors of DVA?
- Why/Why not?
- What do you think the barriers are to GPS/PHCPS's identifying this group?

How successful do you feel the training was in supporting GPS/PHCPSs to identify and support male survivors of DVA?
- Why/Why not?
- What do you think the barriers are to GPS/PHCPS's identifying this group?

How successful do you feel the training was in supporting GPS/PHCPSs to identify and support female perpetrators of DVA?
- Why/Why not?
- What do you think the barriers are to GPS/PHCPS's identifying this group?

How successful do you feel the training was in supporting GPS/PHCPSs to identify and support male perpetrators of DVA?
- Why/Why not?
- What do you think the barriers are to GPS/PHCPS's identifying this group?

How successful do you feel the training was in supporting GPS/PHCPSs to identify and support children exposed to DVA?
- Why/Why not?
- What do you think the barriers are to GPS/PHCPS's identifying this group?

How do you think the IRIS+ training and intervention can be developed to support GPS/PHCPSs in identifying DVA and making referrals to relevant agencies?

<u>Now thinking about your role as an AE …</u>

<u>Referral and first contact</u>

Can you give a rough estimate of how many:

- Women have been referred to your service by GPs/HPs in the last (xx) months?
- Men have been referred to your service by GPs/HPs in the last (xx) months?
- Children have been referred to your service by GPs/HPs in the last (xx) months?

Have the numbers of referrals been what you expected? What surprised you? (if anything).

Can you describe how the referral process from the GP's/PHCPS to your service works?

Are the referrals appropriate?

Variation between different groups of PHCPs and different practices (including IRIS trained and IRIS naïve; size of practices), perceived reasons for this?

What could be improved in relation to the referral process?

From your perspective, what are the barriers to primary HCPs referring to your service? (E*xplore in relation to men/women/victims/perpetrators/children as relevant*)

So far, when you made contact with adults who had been referred to the IRIS+ service:

- How did the initial contact go? (e.g. was adult expecting contact to be made, had they remembered that referral had been made)
- Did referred adults express any concern or annoyance at being referred to your service?
- If adults were not willing to meet with you or did not want further support, what reasons did they give (if any)?
- Were there any particular groups of adults who were not willing to engage further with your service? Why do you think that is?

Have you worked with the safeguarding lead in each practice or someone else?

Have you worked with clinicians in any particularly difficult or challenging situations?

Do you talk to clinicians over the telephone?

How often have you visited the different practices. Is it helpful doing so? Any challenges in relation to this?

Do you think having male and female victims and perpetrators to your service was successful? Why/why not? *(ask if appropriate)*

Support work

I want now to turn to the kind of support that your service offers to adults and/or children, what that support looks like, how it was received by IRIS+ clients and how you think it could be improved.

Thinking about your work as an Advocate Educator for the IRIS+ study, what support have you offered and provided so far for (*ask as appropriate*):

- Women who are victims of DVA
- Women who are perpetrators of DVA
- Men who are victims of DVA
- Men who are perpetrators of DVA
- Children exposed to DVA

Of those groups, (women victims/perps, male victims/perps, children) which do you think your service is particularly good at supporting and working with? Why?

Do you think your service needs to develop and improve its support to any of these groups? (women victims/perps, male victims/perps, children). How could this be done?

Have IRIS+ clients asked for support with things you could not help them with?

- If yes, can you give me some examples?
- What did you do in these situations (e.g. did you refer them on to other services etc)

Thinking about your work as an Advocate Educator for the IRIS+ study, do you see clients most often:

- For a one off appointment
- For regular appointments

How do you think adults and children responded to the support offered by your service?

What impact (both positive and negative) do you think your support is having on the lives of adults and children you have worked with?

Looking forward

How do you think your support service needs to develop to meet the needs of adults and children being referred by primary healthcare professionals?

**How do you think your support service needs to develop for IRIS+ to be rolled out moving forward?**<br><br>**What could we do to improve the referral process from GPs and for other primary healthcare professionals?**

Conclusion

Let the participant know that it's the end of your questions and ask them if there are any other comments that they would like to make.

Thank participant for their time.
