# Supplementary material for: Feasibility of a reconfigured domestic violence and abuse training and support intervention responding to affected women, men, children and young people through primary care
Source: BMC Prim Care. 2024 Jan 26;25:38. doi: 10.1186/s12875-023-02249-5 (PMC10811857; doi:10.1186/s12875-023-02249-5)
Supplement: Supplementary file 8 — Additional file 8. [file 12875_2023_2249_MOESM8_ESM.pdf]

Study to test the feasibility of a training and support intervention for general practice to improve the response to women, men and children exposed to domestic violence and abuse (DVA) – Stage 2

Adult IRIS+ clients/patients – baseline plus COVID questions

Topic guide*

Introductory statement

Thank you for agreeing to do this interview. Today I would like to ask you some questions about your experiences of seeing/speaking to the GP [or other PCHP] and being referred to…The interview will last up to an hour. If there are any questions that you don't feel comfortable answering, just tell me and I'll move on to another topic. Or, if you decide you want to stop the interview altogether that's fine, just let me know. Our conversation today is completely confidential.

We are interested in hearing about your views and experiences. Although I have a list of questions, please do mention anything that you think is important that I don't ask you. If anything is unclear during the interview, let me know.

Consent checklist

Check participant:

- *Has read the participant information sheet*
- *Understands that their participation is voluntary and that they can change their mind and withdraw at any time without having to give a reason and this won't impact on the support that they are receiving from the IRIS+ service.*
- *Understands that if I have serious concerns about their safety, or that of any children they mention, that I may need to share this concern with an appropriate agency.*
- *Understands that personal information about them (such as my name and address) will be treated with strict confidence and securely stored separately from all other data about them (e.g. interview transcripts) at the University of Bristol.*
- *Agrees that the anonymised information collected about them (anonymised transcripts) may be used to support the current research and relevant future research and may be shared anonymously.*

Any questions?

Consent for recording

With your permission, I'd like to digitally record the interview. This is so the interview can be transcribed. It will be erased after being transcribed. All names of people or places which might identify you or others will not be transcribed. Are you happy to continue with the interview and for it to be digitally recorded?

The recording of the interview will be kept securely and only the anonymised transcript of that interview will be used within the research. The recordings themselves will be erased after transcription.

Can I just confirm that I have your consent to be audio-recorded while this interview takes place?

I'll turn the recorder on now then, and for the record state:

Today's date is…… my name is……… and your name is ……… and I have your consent to record this interview? (yes)

Background/context of help-seeking and experience/impact of COVID-19 lockdown measures

- Reflecting back, can you tell me about your visit to the GP and what prompted you to make an appointment with them?

If the GP contact was post-COVID-19 lockdown measures:

- How was the process of getting an appointment and the appointment itself impacted by the COVID-19 lockdown measures?

→ **Prompts to be used if needed:**

- Did the lockdown measures prompt/prevent help-seeking or make no difference?
- Prior knowledge of GP surgery remote operating?
- Any difficulties getting an appointment?
- Where did you get information on how the GP surgery was operating i.e. website; Facebook page, other?

- Can you briefly describe how the COVID-19 lockdown measures have impacted you and your family practically?

→ **Prompts to be used if needed:**

- Living context such as housing, family contact
- Employment and/or access to benefits
- Relationship including with current/ex-partner or abuser and children (if any)

Referral process to IRIS+ and previous support

Explore the referral process in chronological order where possible:

If the GP appointment was made during COVID-19 lockdown measures:

- Can you tell me about the format of the GP / health care professional consultation?

→ **Prompts to be used if needed:**

- Feelings about having a phone/video call versus in-person consultation?
- Feeling about speaking about the abuse over the phone/on video call?
- Were you alone when you talked to GP?
- Did the GP follow it up with you? (Have you been in touch with your GP, or have the GP contacted you since the referral?)

All clients:

- Can you recall who it was who talked to you (e.g. GP, Nurse, HV etc) about your relationship difficulties and the possibility of referral to IRIS+?
- Can you tell me a little bit about what happened during this conversation/visit to [X] health professional?
- What made you decide to talk to that [primary healthcare professional] on that day?
- Have you seen the same [primary healthcare professionals] before?

If the person has seen this GP/healthcare professional before:

- Was it important to you to talk about these difficulties with a [primary healthcare professionals] you knew? (If relevant)

And, if this consultation was during the lockdown period:

- Continuity of care with clinician was more/equally important as before lockdown measures (if relevant)?

All clients:

- On that day, what did you think about the [primary healthcare professionals] initial response to your situation?
- What did you expect from the referral?

- How long until first contact with AE?
- What was the means of communication?
- What was the process of being referred to … support service like for you?

→ **Prompts to be used if needed:**
- Some people have talked of feeling anxious or hopeful about having support?
- If anxious, what in particular were you worried about?

- Who explained the … support service to you? How clearly/well did they explain it?

→ **Prompts to be used if needed:**
- Was anything confusing?
- Would anything else have helped you to understand it more clearly?
- Did you feel able to ask questions? Why? Why not?

- How would you have felt about contacting … support worker (or any other support agency) yourself if the GP/HCP had not referred you?

→ **Prompts to be used if needed:**
- Would you have done it?
- Would you have referred yourself if they had given you a card with a number?
- If they had shown you a website with details?

- Have you talked about these difficulties/issues to do with your relationships before [primary healthcare professionals] or to any other nurse or GP or anyone else at the practice?

If the client has talked about their relationship difficulties with anyone else:
- Who else had you talked to?
- Why had you felt able to talk to this person/people? How did they respond?
- How openly were you able to talk about these issues?
- What helped/stopped you talking about these issues?
- Ever talked to family or friends about these issues?
- How did they respond?

If the client has not talked about their relationship difficulties with anyone else:
- Can you tell me a little bit about why you have not spoken to anyone else previously?

All clients:
- Could anyone have helped you and your family earlier?
- Were there missed opportunities for help do you think?

Work with IRIS+

- Have you already met the … worker? (How many times?)
- Have you seen the AE before the lockdown and afterwards?

If the AE contact occurred before and after COVID-19 lockdown measures:
- What are the main differences pre- and post-covid measures [format, frequency, duration, how they feel about this change, preferences]
- What are the issues with remote IRIS+ support can address effectively and what are the issues that are impossible to address ‘remotely’. How is emotional support via phone?

If the AE contact occurred before and after COVID-19 lockdown measures:
- What are the main differences pre- and post-covid measures [format, frequency, duration, how they feel about this change, preferences]

- What are the issues which remote IRIS+ support can address effectively and what are the issues that are impossible to address 'remotely'. How is emotional support via phone?

If the client has met the AE face-to-face pre-lockdown:

- Where did you meet them?
- Is this a good location for you?
- Has travelling to the location been acceptable?

If the client has not seen/spoken to the AE regularly:

- How often do you think you are you going to see the …worker?
- How often would you like to see the … worker?

If the client still has contact with the perpetrator:

- Has your partner/child been aware of you meeting the … worker?
- How do you feel about this/what do they think?

All clients:

- How are you getting on so far?
  → **Prompts to be used if needed:**
  - Does s/he feel like someone you can trust?
  - What makes you feel this way (if does/does not trust)?

If the client is male:

- What are your thoughts about having a female AE?

All clients:

- Thinking back to before you met your support worker:
  → What did you hope the support worker could help you with?
  → Do you think that they are helping you with this? How? Why/why not?

COVID-19 social distancing measures specific impact on health (mental and physical) and experience of abuse

If not already covered explore the following:

- How have you/are you dealing with stress, physical confinement, self-isolation.
- Can you tell me a little about how COVID-19 and social distancing measures have/are impacting on your health and wellbeing including mental health and feelings about historical/current abuse [probe changes over the time].
- Has the abusive behaviour changed as a result of self-isolation (if relevant) [increased/decreased, intensity, frequency, type, family involvement].
- In general do you feel more or less safe during social distancing measures period.
- Have you had to seek additional support from their primary health care professionals and/or from the IRIS+ service, or were offered any?
- Do you know where to get support if needed.
- Their views on the impact of social isolation measures on family life and other family members living in the household? Are any of your children getting support from IRIS+ or from other services? If yes, describe format, content and impact…
- How do feel about the future and the easing of social distancing/covid lockdown measures?

Final points

- Thinking about your experiences so far of being referred to and supported by …, are there any ways you think the support from the [primary healthcare professional you saw] or … could be improved?

- Is there anything else you think is important for us to know about your experiences of being involved in the study?
- Are you happy for the recording of the interview to be kept?
- Thank you for your time. Explain that will contact them again for follow up interview, probably around [X time].

*Please note this is a suggested guide for interviews only. In keeping with standard practice in qualitative research, these questions may be modified & added to as the trial progresses and new themes or areas of interest emerge. We will also adapt this guide for use with participants at earlier or later stages of the study.
