# Supplementary material for: Feasibility of a reconfigured domestic violence and abuse training and support intervention responding to affected women, men, children and young people through primary care
Source: BMC Prim Care. 2024 Jan 26;25:38. doi: 10.1186/s12875-023-02249-5 (PMC10811857; doi:10.1186/s12875-023-02249-5)
Supplement: Supplementary file 9 — Additional file 9. [file 12875_2023_2249_MOESM9_ESM.pdf]

# Study to test the feasibility of a training and support intervention for general practice to improve the response to women, men and children exposed to domestic violence and abuse (DVA) – Stage 2

**Adult IRIS+ clients/patients – follow up plus COVID questions**

**Topic guide***

## Introductory statement

Thank you for agreeing to do this interview. In this interview I would like to ask you some questions about your experiences of receiving support from IRIS+ [name of agency]. The interview will last between 20-30 minutes. If there are any questions that you don't feel comfortable answering, just tell me and I'll move on to another topic. Or, if you decide you want to stop the interview altogether that's fine, just let me know. Our conversation today is completely confidential.

We are interested in hearing about your views and experiences. Although I have a list of questions, please do mention anything that you think is important that I don't ask you. If anything is unclear during the interview, let me know.

## Consent checklist

*Check participant:*

- *Has read the participant information sheet*
- *Understands that their participation is voluntary and that they can change their mind and withdraw at any time without having to give a reason and this won't impact on the support that they are receiving from the IRIS+ service.*
- *Understands that if I have serious concerns about their safety, or that of any children they mention, that I may need to share this concern with an appropriate agency.*
- *Understands that personal information about them (such as my name and address) will be treated with strict confidence and securely stored separately from all other data about them (e.g. interview transcripts) at the University of Bristol.*
- *Agrees that the anonymised information collected about them (anonymised transcripts) may be used to support the current research and relevant future research, and may be shared anonymously.*

*Any questions?*

## Consent for recording

With your permission, I'd like to digitally record the interview. This is so the interview can be transcribed. It will be erased after being transcribed. All names of people or places which might identify you or others will not be transcribed. Are you happy to continue with the interview and for it to be digitally recorded?

The recording of the interview will be kept securely and only the anonymised transcript of that interview will be used within the research. The recordings themselves will be erased after transcription.

Can I just confirm that I have your consent to be audio-recorded while this interview takes place?

I'll turn the recorder on now then, and for the record state:

**Today's date is…… my name is……… and your name is ……… and I have your consent to record this interview? (yes)**

| **Work with IRIS+/impact of COVID-19 lockdown measures** |
|---|
| *All clients:*<br>• How often have you seen/do you see your IRIS+ worker?<br>• Have you seen the AE before the COVID-19 lockdown measures and/or during them?<br><br>*If the AE contact occurred before and after COVID-19 lockdown measures:*<br>• What are the main differences pre- and post-covid measures [format, frequency, duration, how they feel about this change, preferences]<br>• What are the issues which remote IRIS+ support can address effectively and what are the issues that are impossible to address ‘remotely’. How do find emotional support via phone?<br><br>*If the client has met the AE face-to-face pre-lockdown:*<br>• Where do/did you meet them?<br>• Is this a good location for you?<br>• Has travelling to the location been acceptable?<br><br>*If the client still has contact with the perpetrator:*<br>• Has your partner/child (if appropriate) been aware of you meeting the IRIS+ worker?<br>*All clients:*<br>• How have you been getting on since we last spoke in [month]?<br>→ **Prompts to be used if needed:**<br>- Does the AE/SCW feel like someone you can trust?<br>*If the client is male:*<br>• What are your thoughts about having a female AE?<br>*All clients:*<br>• Thinking back to before you met your support worker:<br>o You told me when we last spoke that you hoped that your support worker would be able to help you with [insert text]?<br>o Has your support worker been able to help you with these things?<br>o Has your support worker been able to help you with anything else?<br><br>• What have you found most useful about having a … support worker? Can you give examples?<br><br>• What have you found most difficult/challenging about having a … support worker? Can you give examples?<br>If the client has children 18 years and under living at home:<br>• Have they been offered support?<br>*If children were offered help:*<br>• What type of support and from whom?<br>• Was this taken up? Why? / Why not? How useful has it been?<br>*If not offered help for the children:*<br>• Would it have been useful? Why?<br>• Have you tried before to get help for your children? From whom?<br>• What was the reaction?<br>*All clients:*<br>• Are there any ways you think the support from … could be improved? |

| **Trajectory since referral to IRIS+** |
|---|

*All clients:*

- How would you describe your relationship with your partner / ex-partner and your family [children?] before you had a support worker with …?
  - → Have those relationships changed at all since you have had a support worker?
  - → How would you describe them now?
  - → How do you think you ex/partner / family/ children would describe your relationship both in the past and now?
  - → [If changed – why do you think this is?]

- Has anything else significantly changed in your life [in the time period of having a referral to IRIS+ that has affected your relationships?

**COVID-19 social distancing measures specific impact on health (mental and physical) and experience of abuse**

*If not already covered at baseline or during follow up interview explore the following:*

- How have you/are you dealing with stress, physical confinement, self-isolation.
- Can you tell me a little about how COVID-19 and social distancing measures have/are impacting on your health and wellbeing including mental health and feelings about historical/current abuse [probe changes over the time].
- Has the abusive behaviour changed as a result of self-isolation (if relevant) [increased/decreased, intensity, frequency, type, family involvement].
- In general do you feel more or less safe during social distancing measures period.
- Have you had to seek additional support from their primary health care professionals and/or from the IRIS+ service, or were offered any?
- Do you know where to get support if needed.
- Their views on the impact of social isolation measures on family life and other family members living in the household? Are any of your children getting support from IRIS+ or from other services? If yes, describe format, content and impact…
- How do feel about the future and the easing of social distancing/covid lockdown measures?

**Final points**

*All clients:*

- Do you have a strategy for getting support in the future if things get difficult in your relationships?
  **Prompt to be used if needed**
  - What do you think you will do / who will you talk to?

- Is there anything else you think is important for us to know about your experiences of being involved in the study?

- Are you happy for the recording of the interview to be kept?

- Thank you for your time.

*Please note this is a suggested guide for interviews only. In keeping with standard practice in qualitative research, these questions may be modified & added to as the trial progresses and new themes or areas of interest emerge. We will also adapt this guide for use with participants at earlier or later stages of the study.
