# Supplementary material for: Feasibility of a reconfigured domestic violence and abuse training and support intervention responding to affected women, men, children and young people through primary care
Source: BMC Prim Care. 2024 Jan 26;25:38. doi: 10.1186/s12875-023-02249-5 (PMC10811857; doi:10.1186/s12875-023-02249-5)
Supplement: Supplementary file 10 — Additional file 10. [file 12875_2023_2249_MOESM10_ESM.pdf]

Study to test the feasibility of a training and support intervention for general practice to improve the response to women, men and children exposed to domestic violence and abuse (DVA) – Stage 2

IRIS+ children/YP (8-12 years): Consent and interview schedule

The interview is semi-structured and will be matched to the maturity, understanding and needs of the child/young person. The questions and statements in this interview schedule should be treated as a guide, rather than an exact replica of what will be said and asked.

Introductory statement

Thank child for agreeing to talk to you today and introduce self.

Explain that you would like to ask them some questions about what it has been like for them to have support from the IRIS+ children's worker, what they have liked and what hasn't been so good.

Go through and talk about the consent form and information sheet. Highlight the following issues:

- Explain why we are doing the research.
- Explain the 'child protection policy' – I would not tell anyone else what they say unless they say something which makes me worry that they, or someone else was going to be hurt. Provide examples or clarify if needed.
- Explain how the information they tell us will be used in the research, how it will be kept safely, how it will be anonymised (give example of what anonymised means)
- Explain that we will talk with each other for around 15-20 minutes but they do not have to answer questions if they don't want to. They just need to say and you will move on to talk about something else.
- If they don't want to take part in the interview any more that's fine and they can stop whenever. If the interview stops or they don't want to answer some questions that is fine. They will still receive the same service from … whatever happens. Give child/young person ways to opt out of answering question e.g. a red card, card which says 'no', a safe topic to start talking about e.g. a pet, a hobby.
- Emphasise that there are no 'right' or 'wrong' answers.
- No-one will be told what they say during the interview, including their support worker/parent/carer.
- Ask the child/young person if they have any questions or if there is anything they are not quite sure about or not happy about.
- Ask the child if they would like mum/dad/other carer present during the interview (*CYP will have already been asked this so this is a confirmation/check in*).

Audio-recording: Ask child if they are okay for the interview to be audio-recorded. Explain that you would like to do this so that you can remember what they say. Tell them it's okay if they don't want to be recorded and you will write notes of what they tell you instead (but you won't write down their name or things that could identify them).

If they are happy to be recorded go through what will happen with the audio after the end of the interview (e.g. transcribed and then deleted; transcript/notes kept in locked cabinet). Show them the digital recorder/what it does/how people sound on it.

Consent process: If the child is willing to go ahead then talk through the signing/initialling of the consent form. *Whether the child is competent to consent (rather than assent) to the interview will depend on the views of the safe parent/carer and the interviewer's perception of the child and how information in the sheet is understood. In either case agreement will be sought from the child and recorded (audio and written).*

Talk to and agree with the young person about how they would like to undertake the interview e.g. talking and answering questions, drawing, writing responses.

If applicable, start audio:
Once agreement has been made about how interview will be recorded, then re-seek consent for the interview which will either be recorded on the digital recorded or in notes.

Conversation starters / 'Getting to know you'

- How old are you? Brothers or sisters? Pets? What do you like doing outside of school?

  Possible activities

  Draw YP in middle of piece of paper and have bubbles coming out which describes YP.

  Do a 'one page profile' (used in person centred approaches) – My name, my age, What are my hobbies and favourite things?

Introduction to topic

Today I want to know about what it's been like getting support from [name of support worker]

- When was the last time you saw (support worker)/went to…?
- How many times have you seen (the support worker)/been to …?
- Where do you meet (the support worker)? *(this may be at …or in the community e.g. a park, at school)*
- How do you feel about speaking to (the support worker) over the phone?
- Would you have liked to meet in person?
- Do you see (the support worker) by yourself or with someone else like your mum/dad/other carer?

Impact of covid measures

- How was it for you when you had to stop going to school and stay at home to protect yourself from covid-19?

Experience of receiving support

Now I want to move on and ask you about your experience of seeing [name of support worker]

- What kinds of things to you do with (the support worker)?
- Can you tell me what kind of things do you talk about with (the support worker)?
- How do you get on with (the support worker)?
- Is (the support worker) friendly?
- Do they listen to you and take what you say seriously?
- Have you ever seen someone like (support worker) before? *(If yes, then this is an opportunity for interviewer to ask some questions about the similarities and differences between what they had before and what they have now)*

Impact of support work (optional – depending on capacity of CYP*)*

- Do you talk to (the support worker) about things that you wouldn't tell other people? Do you think you could talk to (the support worker) about things you wouldn't tell other people like your teacher/ mum/dad? *(This is an opportunity to expand on who the child likes and doesn't like talking to and who the child would see as someone they could talk to e.g. teacher, GP, social worker)*
- Has having contact with (the support worker) made you feel any different (e.g. happier, sadder, angry) at home? In school? With your friends? *(depending on level of understanding/engagement with interview this may be an opportunity to develop an understanding of how things may have changed at home before and after the engagement with support worker)*
- Is there anything you don't like about [support worker]? Are there things that are difficult to talk about with [support worker]? *(this is an opportunity to develop e.g. do they dislike going to the …, do they feel different because they have a support worker when friends or siblings might not)*

Looking ahead

- Would you recommend the IRIS+ support to other children like you? What makes you say that?

Conclusions/wrapping up

- Is there anything else you want to tell me about having a support worker or anything else you think is important for me to know?

If appropriate, briefly summarise what the child/young person has said during interview.

Depending on age and understanding of the child, a follow up question may be asked about how the child found the interview and whether any questions were particularly difficult or easy to answer.

Ending

Thank child for their contribution. Remind them that the interview is confidential and information is kept safely. Ask the child if they have any questions?

Provide contact details if child has questions in future. Confirm researcher/project will keep in touch with child as to the outcomes of the research and check in with child how they would like to be contacted.

Thank you voucher. If it is appropriate, and as a means to finish the interview on a positive note, the following questions could be asked – following up on introductory topics discussed e.g. pets/hobbies/holidays.
