# Supplementary material for: Feasibility of a reconfigured domestic violence and abuse training and support intervention responding to affected women, men, children and young people through primary care
Source: BMC Prim Care. 2024 Jan 26;25:38. doi: 10.1186/s12875-023-02249-5 (PMC10811857; doi:10.1186/s12875-023-02249-5)
Supplement: Supplementary file 11 — Additional file 11. [file 12875_2023_2249_MOESM11_ESM.pdf]

Study to test the feasibility of a training and support intervention for general practice to improve the response to women, men and children exposed to domestic violence and abuse (DVA) – Stage 2

IRIS+ children/YP (13-18 years): Consent and interview schedule

The interview is semi-structured and will be matched to the maturity, understanding and needs of the young person. The questions and statements in this interview schedule should therefore be treated as a guide, rather than an exact replica of what will be said and asked.

Introductory statement

Thank young person for agreeing to talk to you today and introduce self.

Explain that you would like to ask them some questions about [what it has been like for them to have support from IRIS+, what they have liked and what hasn't been so good.]

Go through and talk about the consent form and information sheet. Highlight the following issues:

- Explain why we are doing the research.
- Explain the 'child protection policy' – I would not tell anyone else what they say unless they say something which makes me worry that they, or someone else was going to be hurt. Can provide examples or clarify if needed.
- How the information they tell us will be used in the research, how will it be kept safely, how it will be anonymised (give example of what anonymised means)
- Explain that will talk with each other for around 20-30 minutes but they do not have to answer questions if they don't want to. Just say and you will move on to talk about something else.
- If they don't want to take part in the interview any more that's fine and can stop whenever. If the interview stops or they don't want to answer some questions that is fine. They will still receive a service from IRIS+ whatever happens. Give young person ways to opt out of answering question e.g. a red card, card which says 'no'.
- Emphasise that there are no 'right' or 'wrong' answers.
- No-one will be told what they say during the interview including their support worker/parent/carer
- Ask the young person if they have any questions or if there is anything they are not quite sure about or not happy about.
- Ask the young person if they would like mum/dad/other carer present during the interview. (CYP will have already been asked this so this is a confirmation/check in).

Audio-recording:
Ask young person if they are okay for the interview to be audio-recorded. Explain that you would like to do this so that you can remember what they say. Tell them it is okay if they don't want to be recorded and you will write notes of what they tell you instead (but you won't write down their name or things that could identify them). What would they prefer?

Consent process: If the young person is willing to go ahead then talk through the signing/initialling of the consent form. Whether the young person is competent to consent (rather than assent) to the interview will depend on the views of the safe parent/carer and the interviewer's perception of the young person and how information in the sheet is understood. In either case agreement will be sought from the young person and recorded (audio and written).

Talk to and agree with the young person about how they would like to undertake the interview e.g. talking and answering questions, drawing, writing responses.

If applicable, start audio:
Once agreement has been made about how interview will be recorded, then re-seek consent for the interview which will either be recoded on the digital recorded or in notes.

Conversation starters / 'Getting to know you'

- How old? Brothers or sisters? Pets? What are their interests?

Introduction to topic

Today I want to know about what it's like getting support from IRIS+:

- When was the last time you spoke to/saw (support worker)/went to…?
- How many times have you seen (the support worker)/been to…?
- Where do you meet (the support worker)?
- How do you feel about speaking to (the support worker) over the phone?
- Would you have liked to meet in person?
- Do you see (the support worker)/ go to … by yourself or with someone else like your mum/dad/other carer?
- Do you know how you could contact (the support worker)/ … if you needed to talk to them?
- How did you get to know about the … support services?
- Did you know that these kinds of support services existed before meeting [support worker]?

Impact of covid measures

- How was it for you when you had to stop going to school and stay at home to protect yourself from covid-19?

Experience of receiving support/having a support worker

Now I want to move on and ask you what your experience of having support from IRIS+ is like:

- Can you tell me what kind of things do you talk about or do with (the support worker)?
- How do you get on with (the support worker)?
- Is (the support worker) friendly?

- Does [support worker] listen to you and take what you say seriously?
- Are you able to talk to (the support worker) about things that you wouldn't tell other people? Do you think you could talk to (the support worker) about things you wouldn't tell other people? *(This is an opportunity to expand on who the child likes and doesn't like talking to and who the child would see as someone they could talk to e.g. teacher, GP, social worker)*

- Has seeing (the support worker) made any difference to anything at home? In school? With your friends? *(depending on level of understanding/engagement with interview this may be an opportunity to develop an understanding of how things may have changed before and after the engagement with support worker).*

- Is there anything you don't like about [support worker]? Are there things that are difficult to talk about with [support worker]?

- Has seeing (the support worker) made anything more difficult at home? At school? With friends? How they feel on a day to day basis? *(depending on level of understanding/engagement with interview this may be an opportunity to develop an understanding of how things may have changed at home before and after the engagement with support worker)*

If the YP has specified elements that makes them unhappy or isn't so good about the support worker then these are a good starting point for the next set of questions.

- How does the child feel these issues could be improved?

- What could be better about the service?

Looking ahead/recommendations:

- Should more children have access to someone like (the support worker)? Why?

- How should children be able to find out about having a support worker?

- Would you recommend IRIS+ support to other young people like you? What makes you say that?

Conclusions/wrapping up

- Is there anything else you want to tell me about having a support worker/support from…?
- Is there anything else you think is important for me to know?

If appropriate, briefly summarise what the young person has said during interview.

Depending on age and understanding of the young person, a follow up question may be asked about how the young person found the interview and whether any questions were particularly difficult or easy to answer.

Ending

Thank young person for their contribution. Remind them that the interview is confidential and information is kept safely.<br><br>*Ask the young person if they have any questions.*<br><br>*Provide contact details if young person has questions in future. Confirm researcher/project will keep in touch with them as to the outcomes of the research and check in with them – ask how they would like to be contacted.*<br><br>*Thank you voucher*<br><br>*If it is appropriate, and as a means to finish the interview on a positive note, the following questions could be asked:*<br><br>*What are you looking forward to in the holidays?*
